# Engineered probiotic alleviates ulcerative colitis by inhibiting M1 macrophage polarization via glycolytic reprogramming

**DOI:** 10.1002/btm2.70067

**Published:** 2025-08-29

**Authors:** Chaoqun Lv, Xinyue Hu, Xiang Li, Wen Shi, Wenbo Li, Yan He, Hongqing Li, Jianxi Bai, Zhenxing Li, Zhipeng Wen, Xinxin Liu, Yuanyuan Ai, Jingchao Li, Xiao Chen, Kaijun Liu

**Affiliations:** ^1^ Department of Intensive Care Medicine Daping Hospital, Army Medical University Chongqing China; ^2^ Department of Nuclear Medicine Daping Hospital, Army Medical University Chongqing China

**Keywords:** macrophage, melanin, ulcerative colitis‐engineered probiotics, glycolytic reprogramming

## Abstract

Ulcerative colitis (UC) remains a significant therapeutic challenge due to its complex pathogenesis involving oxidative stress, immune dysregulation, and gut microbiota dysbiosis. Melanin, a natural biopolymer with robust anti‐inflammatory and antioxidant properties, presents a promising treatment avenue for UC. Probiotics, particularly *Escherichia coli* Nissle 1917 (EcN), have gained recognition for their role in restoring gut homeostasis. In this study, we genetically engineered EcN to overexpress tyrosinase (EcN‐T), facilitating the biosynthesis of melanin specifically for UC treatment. The engineered probiotics demonstrated superior therapeutic efficacy compared to either melanin or EcN administered alone, highlighting a synergistic effect. EcN‐T not only exhibited significant capabilities in scavenging reactive oxygen species and restoring gut microbiota but also possessed the characteristic of enhancing gut colonization time, thereby extending the dosing frequency. Moreover, EcN‐T showcased novel mechanisms, such as the restoration of the intestinal mucosal barrier and the elevation of short‐chain fatty acid levels. Additionally, EcN‐T inhibited M1 macrophage polarization through Hypoxia‐Inducible Factor 1‐alpha (HIF‐1α)dependent glycolytic reprogramming, underscoring its immunomodulatory potential. Collectively, these findings provide new insights into the therapeutic potential of EcN‐T for UC treatment, offering a novel strategy that enhances treatment efficacy while potentially reducing side effects associated with conventional therapies.

**Translational Impact Statement**

Research has shown that excessive production of reactive oxygen species (ROS) and dysbiosis of gut microbiota are key issues in ulcerative colitis. In this study, we constructed an engineered tyrosinase probiotic (ECN‐T), which can synthesize melanin at the site of intestinal inflammation. By eliminating ROS, restoring the intestinal mucosal barrier, regulating gut microbiota, increasing short‐chain fatty acid levels, and modulating immune responses, ECN‐T achieves therapeutic effects.

## 1 INTRODUCTION

Ulcerative colitis (UC) stands as the most prevalent chronic nonspecific inflammatory bowel disease (IBD), characterized by mucous or non‐mucous bloody diarrhea. In 2023, the prevalence of UC was estimated to be 5 million cases around the world, and the incidence is increasing worldwide. It is primarily caused by epithelial barrier damage and an imbalance in inflammatory homeostasis.^1^ The pathophysiology of UC remains unclear, involving multiple factors such as oxidative stress, dysregulation of immune responses, alterations in gut microbiota, genetic susceptibility, and environmental influences.^2^, ^3^ During its progression, oxidative stress induces the production of various reactive free radicals and triggers an inflammatory microenvironment accompanied by the release of numerous proinflammatory cytokines. This rapidly activates and recruits inflammatory mediators, leading to further infiltration of other inflammatory cells and ultimately causing severe inflammatory reactions in the colonic epithelium.^4^, ^5^ The chronic and recurrent nature of UC significantly impacts patients' quality of life. Currently, the primary treatment options include 5‐aminosalicylates, thiopurines, biologic agents targeting tumor necrosis factor a (TNF‐α) and integrins, as well as small‐molecule Janus kinase inhibitors, which aim to achieve remission.^6^, ^7^ However, these therapeutic agents are often associated with severe side effects, including diarrhea, osteoporosis, and opportunistic infections.^8^ Therefore, the treatment of UC is still challenging, and new therapeutic strategies need to be explored.

Recent studies have reported the synergistic enhancement of inflammatory disease treatment by probiotics through the decoration of naturally occurring bioactive substances, such as anti‐inflammatory fibrin filaments.^9^ However, it is imperative that the encapsulated anti‐inflammatory substances are able to withstand the challenging conditions of the gastrointestinal tract, including gastric acid and bile salts, and successfully reach the intestine to achieve the desired therapeutic effect for UC.^10^ Inspired by this, an effective strategy would be to directly produce multifunctional antioxidant materials through probiotics, ensuring optimal biocompatibility and intestinal colonization ability. Among these, *Escherichia coli Nissle 1917* (EcN), as a genetically manipulable probiotic, has garnered widespread attention due to its excellent safety profile, remarkable biocompatibility, precise targeting capabilities, and facultative anaerobic characteristics.^11^ EcN has been intensively studied in therapeutic strategies for IBD and tumors, where a key advantage as a bacterial vector lies in its ability to produce exogenous proteins while performing a natural encapsulation function, thereby promoting controlled release of therapeutic agents through its colonization effect.^12^ A key advantage of EcN as a bacterial carrier lies in its ability to produce exogenous proteins while functioning as a natural capsule, facilitating the controlled release of therapeutic agents through its colonization effects.^11^, ^13^ Furthermore, EcN exhibits pronounced probiotic properties, particularly in treating intestinal diseases such as diarrhea and IBD, with a specific focus on UC. It demonstrates antagonistic effects against various intestinal pathogens and modulates the secretion of immune factors in vivo, thereby enhancing the host's immune response.^14^

Melanin, a naturally occurring biological pigment, is formed through a series of enzymatic reactions involving tyrosine or 3,4‐dihydroxyphenylalanine. It has garnered widespread attention due to its biocompatibility, high stability, and functional tunability.^15^ Widely present in natural organisms, including human hair, skin, pupils, and other tissues and organs, melanin can be broken down and metabolized in the body with negligible side effects. Its potent antioxidant, anti‐inflammatory, and anti‐aging activities make it particularly effective in defending against damage caused by reactive oxygen species (ROS).^16^, ^17^, ^18^ Studies have reported that oral administration of melanin can alleviate dextran sulfate sodium (DSS)‐induced colitis by modulating inflammatory cytokines and oxidative stress, maintaining mucosal barriers, and restoring microbiota alterations.^19^ However, the high cost of commercially available biological melanin, primarily extracted from natural pigments, poses a significant obstacle to its large‐scale application.^20^ To address this, microbial synthesis of melanin via tyrosinase has emerged as a promising, economical, and sustainable suitable alternative. Compared to traditional chemical synthesis methods, biosynthesis offers gentle reaction conditions, environmental compatibility, and excellent biocompatibility, making it particularly suitable for large‐scale production.^21^

**SCHEME 1:**
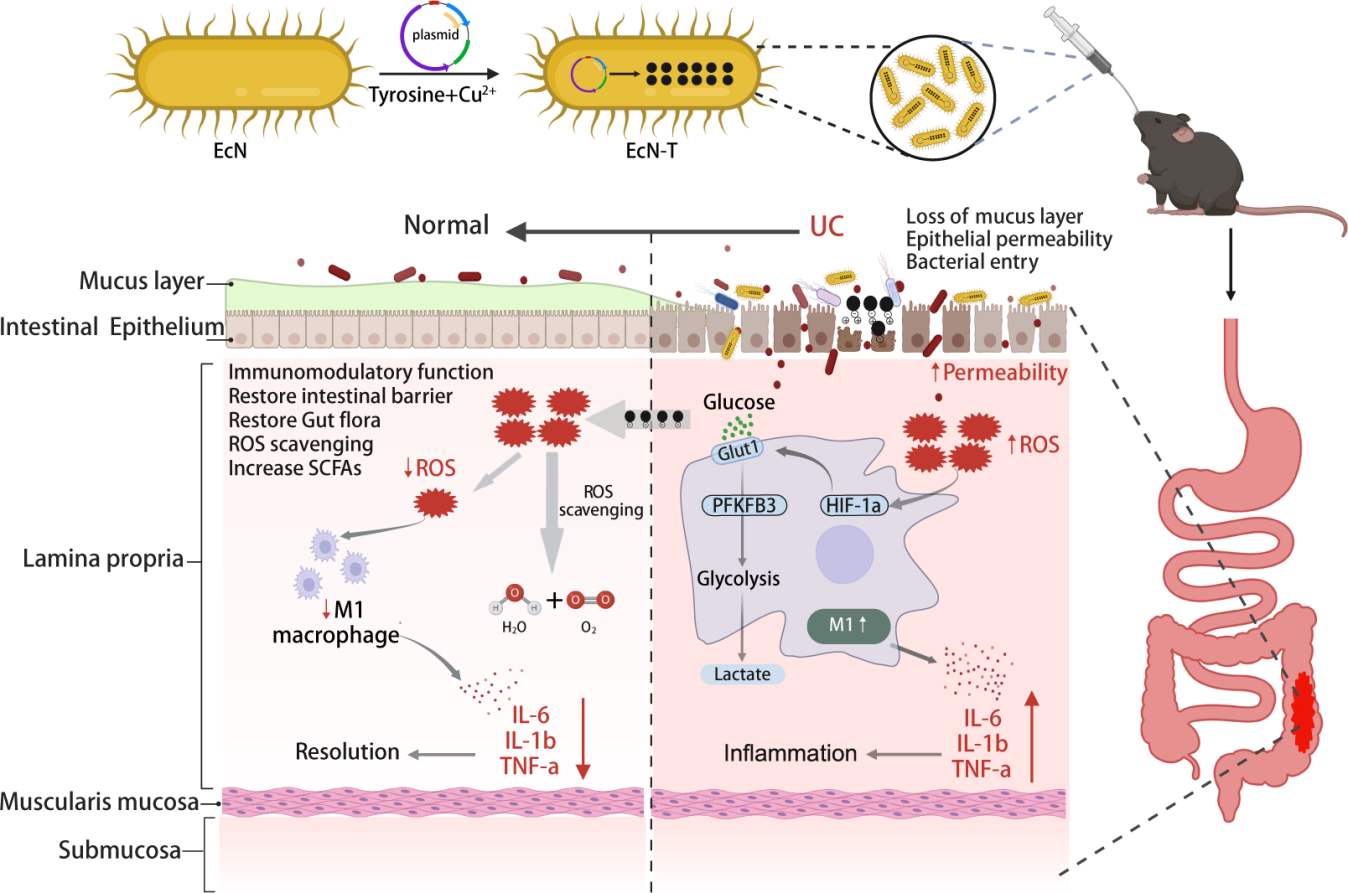
Schematic illustration of EcN‐T‐induced in situ biosynthesis of melanin for ulcerative colitis treatment (created with BioRender.com, with permission). EcN‐T, *Escherichia coli* Nissle 1917‐tyrosinase.

Here, we engineered *Escherichia coli* to overexpress tyrosinase, enabling the production of melanin in vivo (see Scheme 1). Tyrosinase catalyzes a series of biochemical reactions to synthesize melanin, which carries a negative charge and can selectively target positively charged inflammatory colonic lesions through electrostatic interactions, thereby exerting a therapeutic effects.^22^ Experimental results demonstrate that EcN‐T significantly alleviates DSS‐induced colitis primarily through three mechanisms: (i) as a well‐known probiotic, EcN promotes the balance of gut microbiota; (ii) EcN‐T exhibits strong intestinal colonization ability and a longer colonization time, which not only enhances its therapeutic efficacy but also reduces the frequency of dosing; (iii) melanin produced by EcN‐T demonstrates powerful ROS scavenging properties. Notably, EcN‐T outperforms the use of melanin or EcN alone in terms of therapeutic effect, highlighting its synergistic benefits. Furthermore, we untangled new mechanisms by which EcN‐T exerts its effects, including restoring the intestinal mucosal barrier and increasing the content of short‐chain fatty acids. Additionally, EcN‐T inhibits M1 macrophage polarization through HIF‐1α‐dependent glycolytic reprogramming, showcasing its immunomodulatory capabilities. These discoveries provide new insights into the therapeutic potential of EcN‐T in the treatment of UC.

## 2 MATERIALS AND METHODS

### 2.1 Plasmids transformation

EcN and plasmid pCm23119 were purchased from Forhigh Biotech Hangzhou. One microliter of plasmid was added to electrocompetent cells and incubated on ice for 5 min before electroporation at 2500 V. After electroporation, 1 mL of Luria‐Bertani (LB) medium was added, and the cells were incubated at 37°C for 1 h. The culture was spread on LB agar plates containing 34 mg mL^−1^ chloramphenicol, and resulting colonies were inoculated into 100 mL LB liquid medium with chloramphenicol resistance and cultured at 37°C.

### 2.2 Preparation and characterization of EcN‐T

Transformed bacteria were grown overnight at 37°C, then diluted 1:100 into 100 mL LB medium containing 34 mg mL^−1^ chloramphenicol, 50 μg mL^−1^ CuSO_4_·5H_2_O, and 0.8 mg mL^−1^ l‐tyrosine, and incubated for 48 h to produce EcN‐T. The morphology and size of EcN and EcN‐T were characterized using high‐resolution transmission electron microscopy (TEM).

### 2.3 Characterization of melanin extract from melanin@EcN‐T

The pH of the EcN‐T solution was adjusted to 3 with 6 N HCl and allowed to stand at room temperature for 10 h to precipitate melanin. The precipitate was collected by centrifugation, washed, and lyophilized to obtain melanin@EcN‐T powder. The powder was dissolved in ammonia solution, and the ammonia was evaporated to yield melanin@EcN‐T nanoparticles. Scanning electron microscopy (SEM) was used to examine nanoparticle morphology, while dynamic light scattering (DLS) measured hydrodynamic size and ζ‐potential.

### 2.4 Western blotting

Proteins were extracted from intestinal tissues and bacterial cells using Radio‐Immunoprecipitation Assay (RIPA) lysis buffer containing phosphatase and protease inhibitors. The protein concentration was measured using a Bicinchoninic Acid Assay (BCA) protein quantification kit. The membranes were blocked and then incubated overnight at 4°C with specific primary antibodies (GAPDH: 1:1000, 97166T, CST; 6×His: 1:1000, ab237339, Abcam; ZO‐1: 1:1000, ab276131, Abcam; E‐cadherin: 1:1000, TA0131, Abmart; Occludin: 1:1000, ab216327, Abcam; HIF‐1α: 1:1000, D262108, Shanghai Shenggong; PFKFB3: 1:1000, D291137, Sangon Biotech; GLUT1: 1:1000, ab115730, Abcam). The membranes were then incubated with HRP‐conjugated secondary antibodies (Abclonal, Wuhan, China) and developed using a detection kit (Abclonal).

### 2.5 Cell culture

RAW264.7 cells were cultured in Dulbecco's Modified of Eagle's Medium (DMEM) (Gibco, Thermo Fisher, Waltham, MA, USA) supplemented with 10% fetal bovine serum, penicillin, and streptomycin at 37°C in a humidified atmosphere with 5% CO_2_. Cell stimulants included H_2_O_2_ and LPS.

### 2.6 Evaluation of intracellular ROS scavenging ability

H_2_O_2_‐stimulated RAW264.7 cells were incubated with varying concentrations of melanin, followed by 2,7‐dichlorofluorescein diacetate (DCFH‐DA, Solarbio, Beijing, China) staining to assess ROS levels using fluorescence microscopy and flow cytometry.

### 2.7 Animals and grouping

Male C57BL/6 mice were purchased from Vital River Laboratories. Mice aged 6–8 weeks were used for all experiments. The mice were housed and maintained under specific pathogen‐free conditions at the Experimental Animal Center of the Field Surgery Research Institute, Army Medical University. The mice were kept at 22°C with a 12‐h light–dark cycle, with free access to food and water. All animal‐related procedures adhered to the guidelines approved by the Animal Welfare and Ethics Committee of Army Medical University (AMUWEC 20247001). The mice were randomly divided into five groups (*n* = 6): Control group, DSS group, DSS + EcN group, DSS + EcN‐T group, and DSS + Mel. A 3% DSS solution was added to the drinking water for 7 days. Mice received oral administration of EcN (1 × 10^8^ Colony‐Forming Units (CFU)), EcN‐T (1 × 10^8^ CFU), or melanin (100 mg kg^−1^) on specific days, and colon lengths were measured after euthanasia for further analysis.

### 2.8 Reverse Transcription Quantitative Polymerase Chain Reaction (RT‐qPCR)

Total RNA was extracted using TRIzol, and cDNA was synthesized for RT‐qPCR analysis using SYBR Green I (Takara, Kusatsu, Shiga, Japan). The specificity of the PCR products was determined using melt curve analysis, and the relative expression of target genes was calculated using the 2^−(∆∆Ct)^ method (Table S1).

### 2.9 Superoxide dismutase and malondialdehyde detection

Fresh colon tissues were collected, homogenized, and centrifuged to measure malondialdehyde (MDA) concentrations and superoxide dismutase (SOD) activity (Nanjing Jiancheng Bioengineering Institute, Nanjing, China).

### 2.10 Enzyme‐linked immunosorbent assay

Cell supernatants from RAW264.7 were collected and analyzed for pro‐inflammatory (TNF‐α, IL‐6, IL‐1b) and anti‐inflammatory (IL‐10) cytokines using enzyme‐linked immunosorbent assay (ELISA) kits (Abclonal, Wuhan, China).

### 2.11 H&E staining

Colon tissues were fixed in paraformaldehyde, embedded, sectioned, and stained with H&E for microscopic examination.

### 2.12 Immunofluorescence staining

Immunofluorescence was performed to assess the expression of tight junction proteins (ZO‐1, E‐Cadherin, Occludin).

### 2.13 Detection of short‐chain fatty acids content

Short‐chain fatty acids were quantified using gas chromatography–mass spectrometry, following standard curve methods for analysis.^23^, ^24^

### 2.14 Intestinal microbiome analysis

DNA extraction and RNA gene sequencing were conducted as previously described.^25^ Target fragments were amplified by PCR, and the amplification products were purified and recovered using magnetic beads (Vazyme VAHTSTM DNA Clean Beads). The recovered products were quantitatively measured using the Quant‐iT PicoGreen dsDNA Assay Kit, with the quantification instrument being a Microplate reader (BioTek, FLx800). Sequencing libraries were prepared using Illumina's TruSeq Nano DNA LT Library Prep Kit, followed by high‐throughput sequencing. Microbiome bioinformatics were mainly performed with QIIME 22019.4, while the Operational Taxonomic Units (OTU) clustering procedure followed the Vsearch (v2.13.4) pipeline described here (https://github.com/torognes/vsearch/wiki/VSEARCH-pipeline).

Briefly, raw sequence data were demultiplexed using the demux plugin, followed by primer cutting with the cutadapt plugin. Sequences were then merged, filtered, and dereplicated using the functions of fastq_mergepairs, fastq_filter, and derep_fulllength in Vsearch. All the unique sequences were then clustered at 98% (via cluster_size) followed by chimera removal (via uchime_denovo). At last, the non‐chimera sequences were re‐clustered at 97% to generate OTU representative sequences and the OTU table. Representative sequences were aligned with mafft and used to construct a phylogeny with fasttree2. Alpha‐diversity metrics (Chao1, Observed species, Shannon, Simpson, Faith's PD, Pielou's evenness, and Good's coverage), along with beta diversity metrics (weighted UniFrac, unweighted UniFrac, Jaccard distance, and Bray–Curtis dissimilarity) were estimated using the diversity plugin with samples. Taxonomy was assigned to ASVs using the classify‐sklearn naïve Bayes taxonomy classifier in the feature‐classifier plugin against the Silva v132 99% OTUs reference sequences.^26^

### 2.15 Intestinal transcriptome analysis

Total RNA was extracted from the intestine using TRIzol reagent (Takara, Kusatsu, Shiga, Japan), and genomic DNA was removed with DNase I (Takara, Kusatsu, Shiga, Japan). mRNA with polyA tails was enriched from total RNA using Oligo(dT) magnetic beads. The RNA was fragmented to approximately 300 bp using ion shearing. Using RNA as a template, the first‐strand cDNA was synthesized with random hexamer primers and reverse transcriptase, followed by the synthesis of the second‐strand cDNA using the first‐strand cDNA as a template. After library construction, PCR amplification was performed to enrich library fragments, followed by size selection to obtain libraries of about 450 bp. The library was then quality‐checked with an Agilent 2100 Bioanalyzer, and the total and effective concentrations of the library were measured. Libraries with different Index sequences were mixed proportionally based on effective concentration and desired data output. The mixed libraries were uniformly diluted to 2 nM and denatured to form single‐stranded libraries. After RNA extraction, purification, and library preparation, the libraries were sequenced using next‐generation sequencing technology on an Illumina platform with paired‐end sequencing. These procedures were carried out by PANOMIX Biotech. The obtained results were analyzed using RStudio and the Reactome data set.^27^

### 2.16 Measurement of glycolysis using seahorse extracellular flux analyzer

For glycolytic function assessment, RAW264.7 cells were seeded into CellTak‐Coated Seahorse XF 24 cell culture microplates (Agilent Technologies) and cultured for 72 h. One day before metabolic measurements, the probe plate was hydrated with XF Calibrant in a CO_2_‐free incubator, and LPS (300 ng mL^−1^) was added to the cell culture microplates to induce differentiation into an M1‐like phenotype. On the day of the experiment, assay media were prepared according to different detection indicators. For oxygen consumption rate (OCR) assay media: mix 97 mL Seahorse XF DMEM medium, 1 mL Seahorse XF 1.0 M glucose solution, 1 mL Seahorse XF 100 mM pyruvate solution, and 1 mL Seahorse XF 200 mM glutamine solution. For extracellular acidification rate (ECAR) assay media: mix 99 mL Seahorse XF DMEM medium and 1 mL Seahorse XF 200 mM glutamine solution. One hour before measurement, the cells were washed and the culture medium was replaced with the respective assay media. According to the manufacturer's instructions and protocols, cellular OCR or ECAR in RAW264.7 cells treated with oligomycin (1.5 μM), carbonyl cyanide‐*p*‐trifluoromethoxyphenylhydrazone (1 μM), and rotenone and antimycin A (R + A, 0.5 μM) or glucose (10 mM), oligomycin (1 μM), and 2‐deoxyglucose (50 mM) were measured using the Agilent Seahorse Bioscience XF 24 extracellular flux analyzer.

### 2.17 Statistical analysis

All statistical analyses were independently repeated three times, and all results were reproducible. All data were presented as mean ± standard deviation. Statistical analyses were performed using GraphPad Prism 8 (GraphPad Software, San Diego, CA, USA). For statistical comparisons, one‐way ANOVA was used. Statistical differences were defined as ****p < 0.0001, ***p < 0.001, **p < 0.01, *p < 0.05, and ns means no significance.

## 3 RESULTS

### 3.1 Construction of engineered probiotics and characterization of melanin@EcN‐T

**FIGURE 1:**
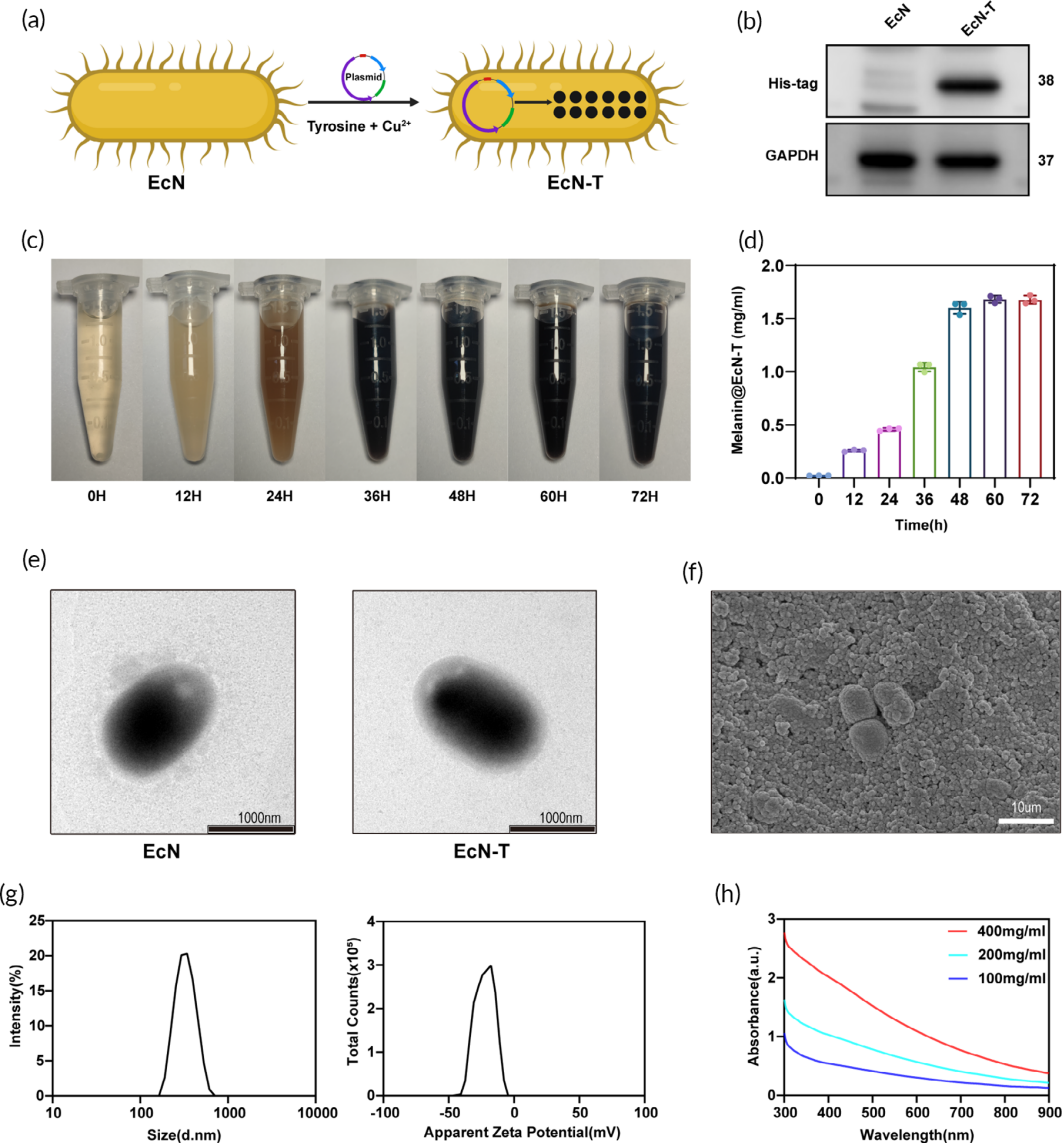
Construction and characterization of the engineered probiotics EcN‐T. (A) Schematic illustration of the biosynthetic process of melanin in EcN‐T. (B) Western blot analyses of 6×His‐tagged tyrosinase in EcN‐T. (C) Pictures were taken at the indicated time points during the biosynthetic process of melanin. (D) Quantitative analysis of melanin yields at different time points (*n* = 3). (E) TEM image of EcN and EcN‐T. Scale bar = 1000 nm. (F) SEM image of melanin@EcN‐T. Scale bar = 10 μm. (G) Hydrodynamic size and zeta potentials distribution of melanin@EcN‐T measured by DLS. (H) The UV–vis–NIR absorption spectra of different concentrations of melanin@EcN‐T nanoparticles (300–900 nm). DLS, dynamic light scattering; EcN‐T, *Escherichia coli* Nissle 1917‐tyrosinase; SEM, scanning electron microscopy; TEM, transmission electron microscopy.

To achieve genetic engineering of EcN‐T, we placed the tyrosinase gene Tyr, which carries a 6×His tag, at the termination of the strong pJ23119 promoter and constructed a recombinant plasmid expressing 6×His‐tagged tyrosinase (Figure S1). By overexpressing tyrosinase in the engineered EcN‐T strain and adding 50 μg mL^−1^ of CuSO_4_·5H_2_O and 0.8 mg mL^−1^ of l‐tyrosine, melanin production was induced successfully (Figure 1a). To verify the expression of tyrosinase, bacteria were harvested and lysed after 24 h of shake flask cultivation. Western blot results confirmed the expression of tyrosinase with an expected molecular weight of 38 kDa (Figure 1b). When CuSO_4_·5H_2_O and l‐tyrosine were added to the culture medium containing EcN‐T and co‐incubated, the color of the medium gradually darkened over time due to the partial release of tyrosinase by the bacteria (Figure 1c). As shown in Figure S2, when both CuSO_4_·5H_2_O and l‐tyrosine are added, the resulting EcN‐T solution appeared significantly darker compared to the EcN solution.

To assess the yield of melanin, we quantified it through absorbance measurements, resulting in a melanin yield of 1.65 mg mL^−1^, which remained constant after 48 h of incubation (Figure 1d). TEM images showed no significant morphological changes in EcN‐T compared to EcN (Figure 1e). SEM was used to characterize the structure of the melanin@EcN‐T nanoparticles, revealing their particle size (Figure 1f). DLS analysis indicated that the melanin@EcN‐T had an average hydrodynamic diameter of 325.4 nm and a zeta potential of −22.4 mV (Figure 1g). The Fourier transform infrared spectroscopy spectra of the melanin nanoparticles showed peaks around at 1250 cm^−1^ (C=O) and 1600 cm ^−1^ (C=C, C=N), indicating the presence of aromatic structures in the melanin (Figure S3).^21^ The optical properties of the melanin@EcN‐T nanoparticles were explored using UV–vis absorption spectroscopy in the range of 300–900 nm (Figure 1h). Electron spin resonance (ESR) spectra displayed the characteristic broad single‐line ESR spectrum, consistent with previous reports (Figure S4).^15^, ^28^ The absorbance of the melanin@EcN‐T nanoparticles showed a concentration‐dependent increase, with a linear regression equation of *Y* = 0.0035*X* + 0.0174 (*R* ^2^ = 0.9977, at 492 nm; Figure S5).

### 3.2 Melanin@EcN‐T alleviates oxidative stress

**FIGURE 2:**
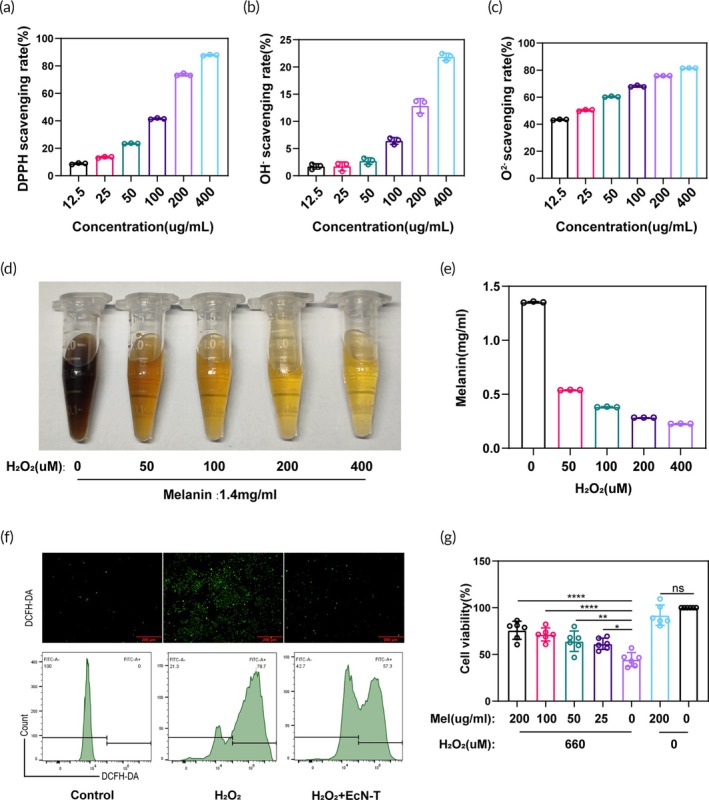
Melanin@EcN‐T alleviates oxidative stress. (a–c) Scavenging efficiencies of DPPH, OH^−^, and ·O^2−^ with melanin@EcN‐T, respectively (*n* = 6). (d) Relative photographic image of solution containing melanin@EcN‐T after the addition of different concentrations of H_2_O_2_. (e) Quantitative results of the remaining melanin after reacting for 24 h with different concentrations of H_2_O_2_ added to a solution containing a certain concentration of melanin (*n* = 5). (f) Fluorescence images of an intracellular ROS scavenging test in H_2_O_2_‐treated RAW 264.7 cells by DCFH‐DA and the corresponding flow cytometry analysis. (g) Cell viabilities of H_2_O_2_‐triggered RAW264.7 cells at the different concentrations of melanin@EcN‐T (*n* = 6). Data are presented as mean ± SD. Statistical comparisons were performed using Mann–Whitney test and unpaired t test. *p < 0.05, **p < 0.01, ***p < 0.001, ****p < 0.0001. DCFH‐DA, 2,7‐dichlorofluorescein diacetate; DPPH, 2,2‐diphenyl‐1‐picrylhydrazyl; EcN‐T, *Escherichia coli* Nissle 1917‐tyrosinase; SD, standard deviation.

Oxidative stress caused by excess ROS is a critical pathological factor in many inflammatory diseases. Our previous studies have demonstrated that melanin exhibits excellent capabilities in ROS scavenging.^29^ To evaluate the antioxidant potential of melanin@EcN‐T, we systematically tested its ability to neutralize various types of ROS, including 2,2‐diphenyl‐1‐picrylhydrazyl (DPPH), hydroxyl radicals (·OH^−^), and superoxide radicals (·O^2−^). As shown in Figure 2a–c, melanin@EcN‐T exhibited potent and broad‐spectrum ROS scavenging activity, with its efficacy increasing significantly in a concentration‐dependent manner. These results confirm that melanin@EcN‐T retains the strong antioxidant properties of melanin, making it highly effective in alleviating oxidative stress. To further investigate the interaction between melanin@EcN‐T and ROS in inflammatory environments, we explored its biodegradation mechanism in the presence of high levels of hydrogen peroxide (H₂O₂), a common oxidative stress signal in UC lesions. As shown in Figure 2d,e, exposure to H₂O₂ caused the UV–vis absorbance of melanin@EcN‐T to gradually decrease, indicating a time and concentration‐dependent degradation. Notably, after reacting with H₂O₂ for 1 day, the high concentration H₂O₂ group exhibited significant fading of nearly all melanin@EcN‐T. This fading is likely caused by the conversion of melanin@EcN‐T into lighter‐colored or colorless oxidation products, such as 5,6‐dihydroxyindole‐2 carboxylic acid. These findings demonstrate that melanin@EcN‐T is not only highly biodegradable but also reacts dynamically with ROS, further amplifying its antioxidative effects in ROS‐rich environments.^30^

To further evaluate the ability of melanin@EcN‐T to scavenge intracellular ROS, we utilized the DCFH‐DA assay kit. Fluorescence imaging and flow cytometry analysis revealed that H₂O₂ stimulation significantly elevated intracellular ROS levels in RAW264.7 macrophages. Remarkably, after incubation with melanin@EcN‐T, intracellular ROS levels were significantly reduced, as demonstrated in Figure 2f. To assess the therapeutic effects of melanin@EcN‐T on inflamed cells, an inflammatory model was established by treating RAW264.7 cells with 660 μM H₂O₂. As shown in Figure 2g, H₂O₂ stimulation led to substantial cell death, with nearly 50% of the cells in the positive control group succumbing to oxidative damage, confirming the successful establishment of the inflammation model. During the modeling process, cells were treated with varying concentrations of melanin@EcN‐T, which resulted in a notable improvement in cell survival rates. Importantly, cell viability increased in a concentration‐dependent manner, with higher concentrations of melanin@EcN‐T providing pronounced cytoprotective effects compared to the positive control group.

These findings underscore the broad‐spectrum antioxidant capabilities of melanin@EcN‐T, particularly its effectiveness in scavenging intracellular ROS and protecting cells from oxidative stress‐induced damage.

### 3.3 EcN‐T can relieve DSS‐induced intestinal inflammation

**FIGURE 3:**
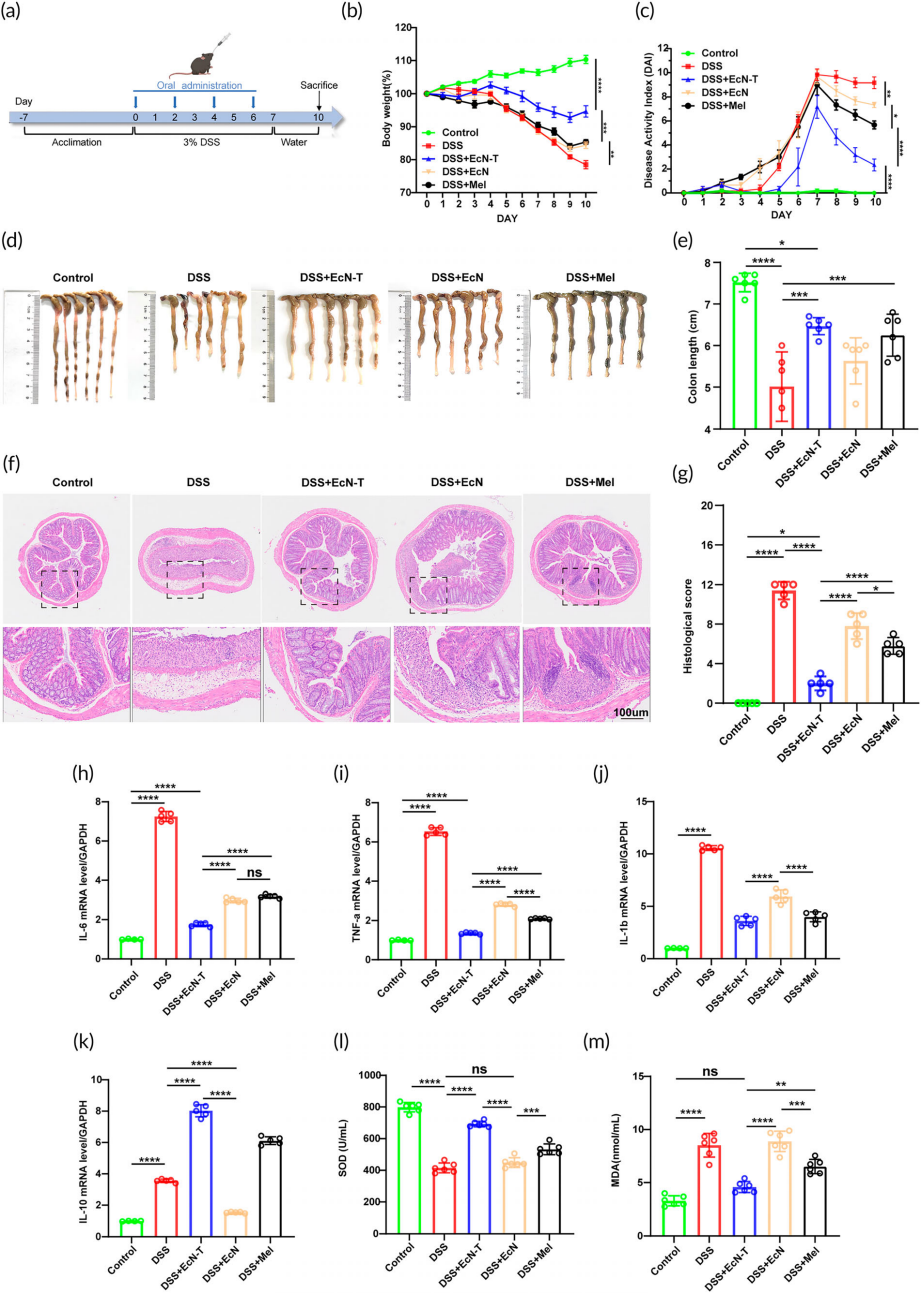
Therapeutic effects of the engineered probiotics EcN‐T in DSS‐induced mouse model. (a) Schematic showing the experimental procedure for the treatment of DSS‐induced UC mice. C57BL/6 mice were given drinking water containing 3% DSS from Day 0 to Day 6. Meanwhile, the mice were fed PBS, EcN, EcN‐T (1 × 10^8^ CFU), or melanin@EcN‐T (200 mg kg^−1^) on Days 0, 2, 4, and 6 by gavage. (b) The body weight of the mice with different treatments. (c) The DAI of mice during the treatment. (d) Photographs and (e) corresponding quantified lengths of colons harvested from mice 10 days after different treatments. (f) Representative images of H&E staining of colon tissue harvested on Day 10 after different treatments. (g) The histological score was calculated to quantify tissue damage. (h–k) The levels of IL‐6, TNF‐α, IL‐1b, and IL‐10 in the colon tissues measured by q‐PCR on Day 10. (l, m) The activities of SOD and the level of MDA in the collected colon tissues. Data are presented as mean ± SD. Statistical comparisons were performed using Mann–Whitney test and unpaired *t*‐test. *p < 0.05, **p < 0.01, ***p < 0.001, ****p < 0.0001. DAI, Disease Activity Index; DSS, dextran sulfate sodium; EcN, *Escherichia coli* Nissle 1917; EcN‐T, *Escherichia coli* Nissle 1917‐tyrosinase; MDA, malondialdehyde; SOD, superoxide dismutase; TNF‐α, tumor necrosis factor α; UC, ulcerative colitis; PBS, phosphate buffered saline; SD, standard deviation.

To evaluate the therapeutic effects of orally administered EcN‐T, we conducted animal experiments using a DSS‐induced UC mouse model. Mice were continuously fed with 3% (w/v) DSS for 7 days to induce acute inflammation, followed by switching to pure water. Starting from Day 0, mice were orally administered EcN, EcN‐T, Melanin, or water every other day, repeating the treatment four times (Figure 3a). Throughout the in vivo study, body weight was measured daily, which is an important indicator for assessing the therapeutic effect of UC. The results showed that the body weight of healthy mice continuously increased over time, while all DSS‐treated groups experienced a decrease in body weight starting from Day 4, indicating the onset of UC. In contrast, the EcN‐T treatment group showed a mitigation in the weight loss trend from Day 5 and an increase in body weight from Day 9 (Figure 3b). The severity of inflammation was further assessed using the Disease Activity Index (DAI) score, which includes criteria such as weight loss, stool bleeding, and stool traits. The results indicated that the DAI scores of the EcN‐T treatment group were significantly lower than those of the other DSS‐treated groups, suggesting reduced inflammation (Figure 3c). Changes in colon length and tissue damage are important indicators for evaluating the therapeutic effects of colitis treatments. The experimental results showed that the colon length of the DSS‐induced colitis mouse model group (5.02 ± 0.83 cm) was significantly shortened compared to the control group (7.52 ± 0.22 cm). In contrast, the colon length of the EcN‐T treatment group (6.47 ± 0.21 cm) was significantly longer than the DSS‐induced groups, indicating that EcN‐T treatment significantly protected mice from DSS‐induced colon shortening (Figure 3d,e). H&E‐stained images of colon tissue revealed that DSS‐induced colitis in mice resulted in significant colon damage, including dilated and congested vessels in the lamina propria, epithelial degeneration and necrosis, mucosal erosion, and diffuse mixed inflammatory cell infiltration in the mucosal lamina propria. In contrast, the colon tissue morphology in the EcN‐T treatment group was similar to healthy mice (Figure 3f). According to histological scoring, EcN‐T treatment restored the integrity of the colonic epithelium and reduced inflammatory infiltration in the mucosa, indicating a normal recovery of colon tissue (Figure 3g).

Next, we measured the levels of pro‐inflammatory cytokines (IL‐6, TNF‐α, and IL‐1β) and the anti‐inflammatory cytokine (IL‐10) in colon tissues using q‐PCR. The results showed that the levels of IL‐6, TNF‐α, and IL‐1β were significantly reduced in the EcN‐T treatment group, while the level of IL‐10 was significantly increased (Figure 3h–k). Finally, to evaluate the impact of EcN‐T on oxidative stress, we assessed SOD activity and MDA content in colon tissues.^31^, ^32^ The results indicated that compared to the control group, the DSS‐induced colitis mouse model group exhibited a significant decrease in SOD activity (Figure 3l) and a significant increase in MDA levels (Figure 3m). The EcN‐T treatment group showed a significant increase in SOD activity and a decrease in MDA levels, with a notable difference compared to the DSS‐induced colitis mouse model group. These results collectively suggest that the EcN‐T treatment group effectively alleviated UC symptoms and performed better than other treatment groups.

### 3.4 Restoration of intestinal mucosal barrier by the engineered probiotics EcN‐T

The intestinal barrier plays a crucial role in maintaining gut health and defending against external pathogens. In a healthy gut, intestinal cells secrete a hydrated gel‐like mucus that acts as a physical barrier separating the intestinal contents from the underlying tissues and regulating the gut microbiota through immunoglobulins and antimicrobial peptides in the mucus.^33^ However, excessive ROS can disrupt the mucus layer, exposing epithelial cells to pathogens and environmental toxins in UC lesions, leading to a loss of intestinal permeability and structural integrity.^3^ Tight junctions are composed of adhesion proteins (such as E‐Cadherin), tight junction transmembrane proteins (such as Occludin), and peripheral membrane proteins (such as ZO‐1).^34^ E‐cadherin initiates the formation of intercellular adherens junctions through intercellular cadherin interactions, ZO‐1 acts as a scaffold protein mediating protein–protein interactions to form connections between adherens and tight junctions, and Occludin regulates cell permeability and adhesion.^35^

**FIGURE 4:**
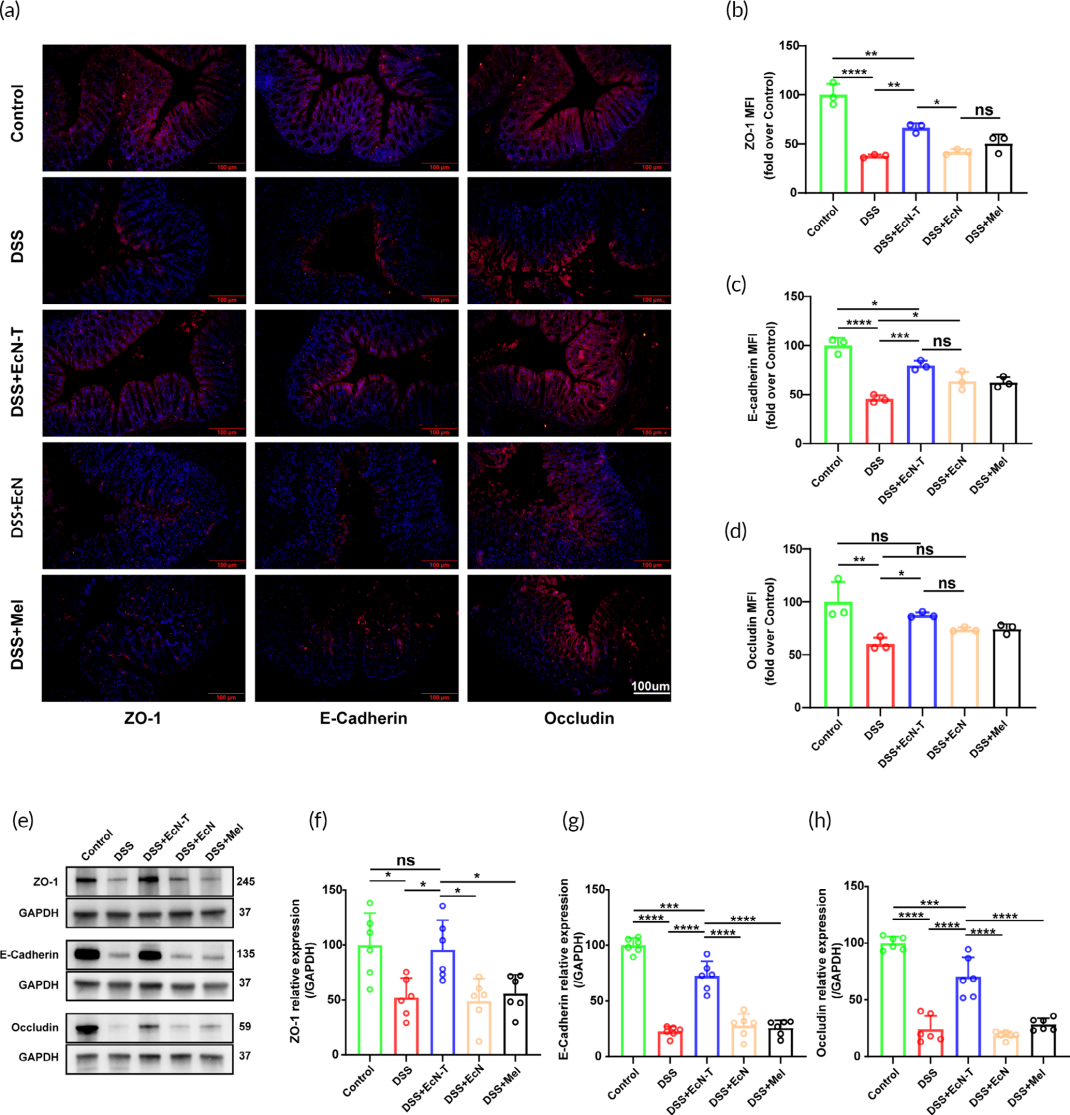
Restoration of intestinal mucosal barrier by the engineered probiotics EcN‐T. (a) Representative immunofluorescence staining images of colon sections to indicate the expression of tight junction proteins, including ZO‐1, E‐Cadherin, and Occludin 10 days after different treatments. Scale bar: 100 μm. (b–d) Mean Fluorescence Intensity of ZO‐1, E‐Cadherin, and Occludin (*n* = 3). (e) Western blot analysis of ZO‐1, E‐Cadherin, and Occludin proteins in colon tissue homogenate from different treatments. (f–h) The q‐PCR quantification results of ZO‐1, E‐Cadherin, and Occludin in colonic tissues (*n* = 6). Data are presented as mean ± SD. Statistical comparisons were performed using Mann–Whitney test and unpaired *t*‐test. *p < 0.05, **p < 0.01, ***p < 0.001, ****p < 0.0001. EcN‐T, *Escherichia coli* Nissle 1917‐tyrosinase; q‐PCR, quantitative polymerase chain reaction; SD, standard deviation.

Immunofluorescence staining and its quantification results showed that in the DSS‐induced colitis mouse model group, the expression of ZO‐1, E‐Cadherin, and Occludin in colon lesion tissues was significantly downregulated. However, in the EcN‐T treatment group, their expression was effectively restored, indicating the recovery of intestinal barrier function (Figure 4a–d). Western blot and q‐PCR analysis further confirmed that EcN‐T treatment increased the expression of ZO‐1, E‐Cadherin, and Occludin in the intestinal tissues. In contrast, the DSS‐induced colitis mouse models orally administered with EcN or melanin did not show similar restorative effects (Figure 4e–h). This evidence strongly demonstrates that EcN‐T effectively restores and maintains intestinal barrier function by promoting the assembly of tight junction complexes.

### 3.5 Regulation of short‐chain fatty acids and gut microbiota by the engineered probiotics EcN‐T

The human gut microbiota, composed of bacteria, fungi/yeast, and viruses, is crucial for health and immune regulation. Changes in its composition are linked to diseases like diabetes, obesity, colorectal cancer, and IBD.^36^ Bacteria that digest dietary fibers produce short‐chain fatty acids (SCFAs) like acetate, propionate, and butyrate, essential for metabolic and immune balance and gut barrier integrity.^37^, ^38^ Current treatments like prebiotics and probiotics are inconsistent, prompting research into next‐generation probiotics with SCFA‐producing bacteria to restore gut‐immune interactions.^39^

**FIGURE 5:**
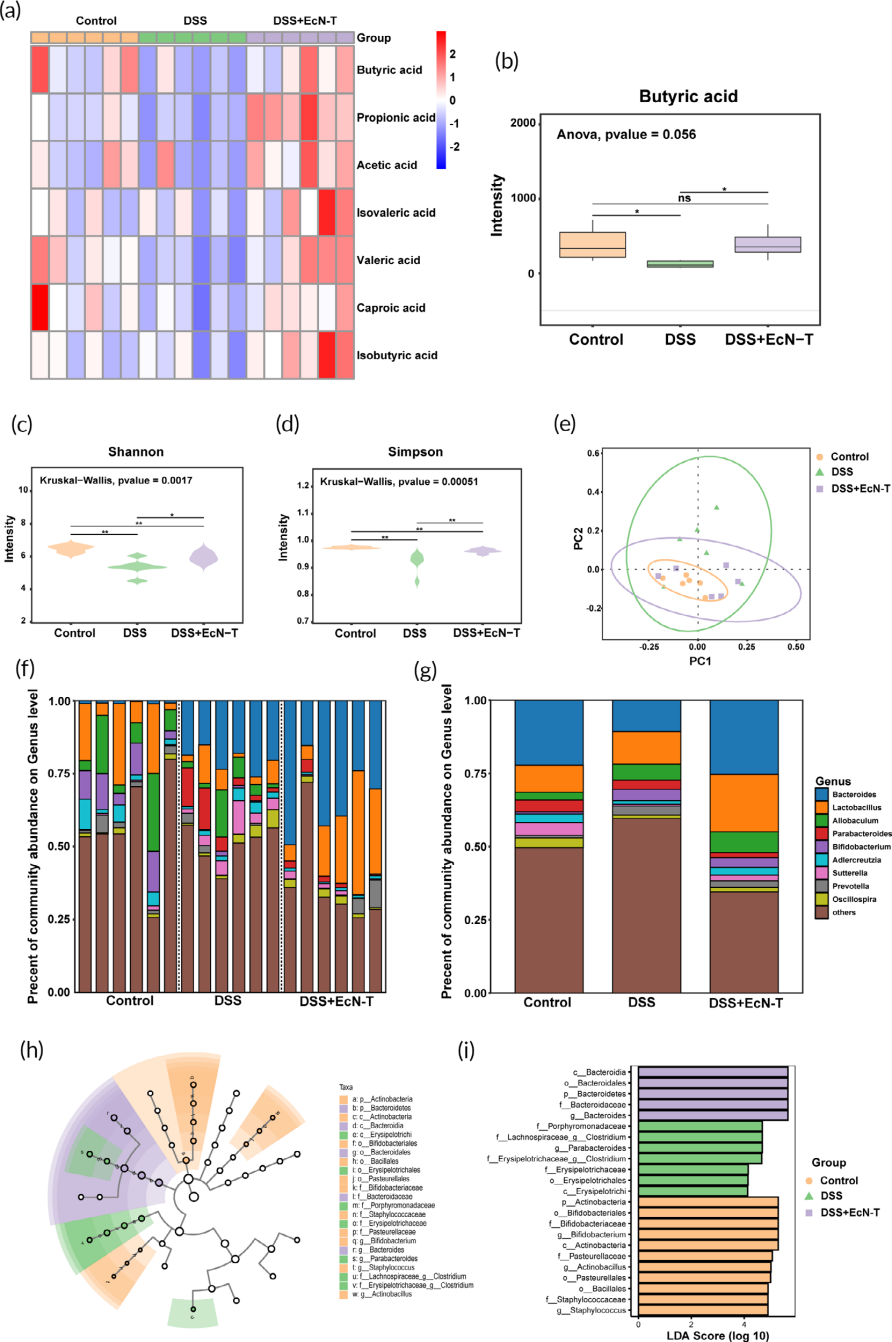
Regulation of SCFAs and gut microbiota by the engineered probiotics EcN‐T. (a) Heatmap of SCFA expression in mice after different treatments (*n* = 6). (b) Quantitative box plot of butyrate expression levels in different treatments (*n* = 6). (c) Shannon index of gut microbiota in mice after different treatments (*n* = 6). (d) Simpson index of gut microbiota in mice after different treatments (*n* = 6). (e) Principal coordinate analysis (PCoA) of different treatments (*n* = 6). (f) Microorganism community structures of the microbiota at the genus level in different treatments (*n* = 6). (g) Community bar plot analysis of the microbiota of mice at the genus level. (h, i) Linear discriminant analysis effect size (LEfSe) analysis of the microbiota in different treatments. Data are presented as mean ± SD. Statistical comparisons were performed using the Mann–Whitney test and unpaired *t*‐test. *p < 0.05, **p < 0.01, ***p < 0.001, ****p < 0.0001. EcN‐T, *Escherichia coli Nissle* 1917‐tyrosinase; SCFAs, short‐chain fatty acid; SD, standard deviation.

SCFAs levels can directly reflect the impact of colitis on mouse metabolism. Figure 5a shows that SCFAs levels significantly decrease in the DSS‐induced colitis model group, while the EcN‐T treatment group restores SCFAs levels, primarily by modulating the levels of butyric acid, valeric acid, and propionic acid to achieve therapeutic effects (Figures 5b, S6, and S7). We further analyzed the gut microbiota in different mouse groups by performing 16S rRNA sequencing on fecal samples. The results showed that the EcN‐T treatment group significantly increased the Shannon and Simpson diversity indices, thereby enhancing microbial diversity (Figure 5c,d). Principal coordinates analysis (PCoA) revealed that, compared to the DSS‐induced colitis model group, EcN‐T intervention significantly impacted the overall microbial composition (Figure 5e). Further analysis of the gut microbiota at the genus level revealed that the EcN‐T treatment group achieved its therapeutic effects by increasing the levels of beneficial bacteria such as *Bacteroides* and *Lactobacillus* (Figure 5f,g).^40^, ^41^, ^42^ Additionally, linear discriminant analysis effect size (LEfSe) showed that the abundance of *Bacteroidia*, *Bacteroidales*, *Bacteroidetes*, *Bacteroidaceae*, and *Bacteroides* significantly increased in the EcN‐T treatment group (Figure 5h,i).^43^ These findings indicate that EcN‐T effectively ameliorates colitis symptoms and restores intestinal homeostasis by modulating SCFAs levels and the composition of the gut microbiota.

### 3.6 EcN‐T could inhibit M1 macrophage polarization via HIF‐1α‐dependent glycolytic reprogramming

**FIGURE 6:**
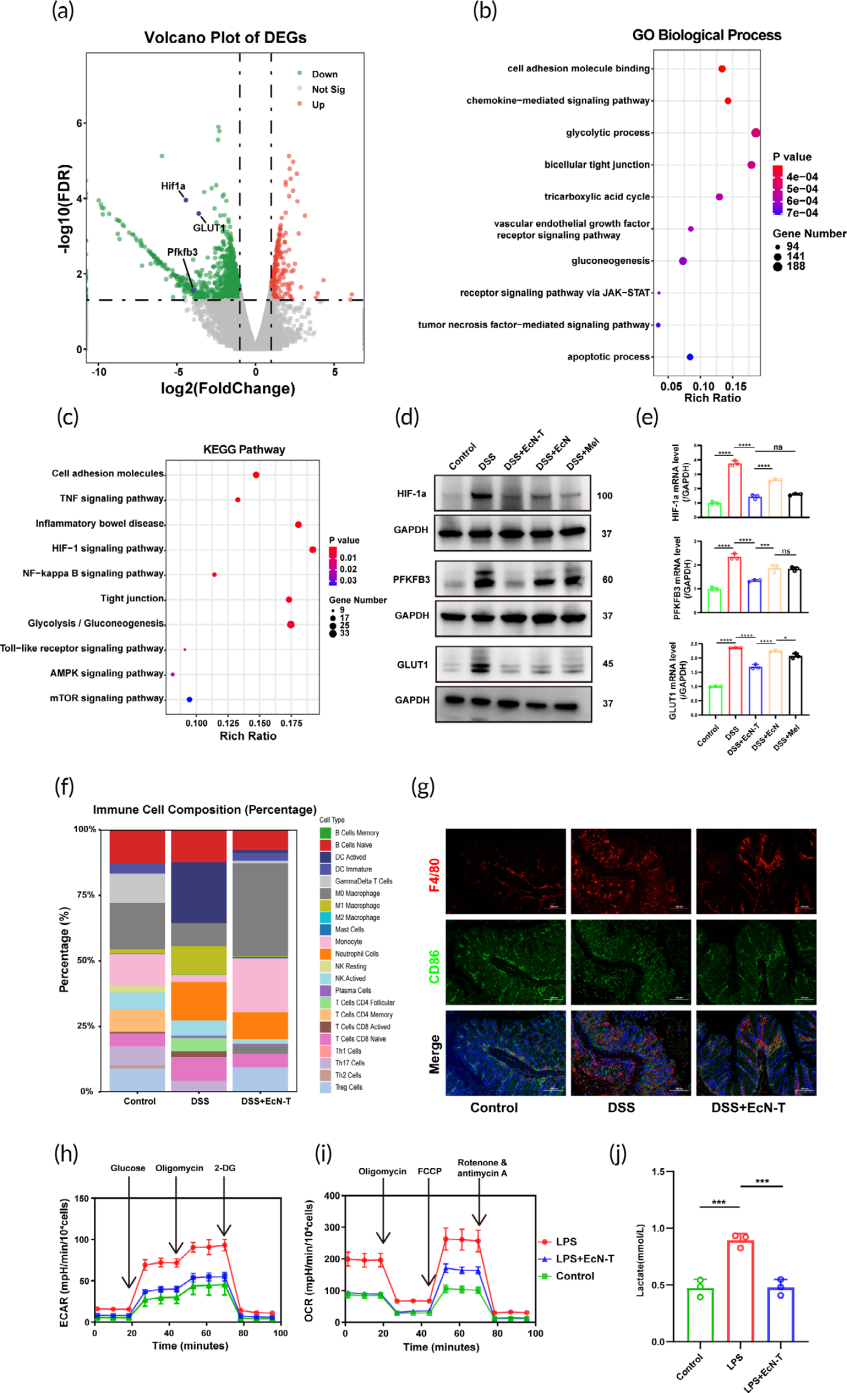
Transcriptomic analysis and investigation of the therapeutic mechanisms of the engineered probiotics EcN‐T. (a) Volcano plot of differentially expressed genes determined between DSS/DSS + EcN‐T (*n* = 4). (b) Gene ontology enrichment analysis of differential genes in DSS and DSS + EcN‐T group (*n* = 4). (c) KEGG pathways enrichment analysis of differential genes in DSS and DSS + EcN‐T group (*n* = 4). (d) The Western blot results of HIF‐1α, PFKFB3, and GLUT1 from different treatments (*n* = 3). (e) The q‐PCR quantification results of HIF‐1α, PFKFB3, and GLUT1 from different treatments (*n* = 3). (f) Using CIBERSORT for immune infiltration analysis results. (g) Immunofluorescence staining of CD86 (M1 marker) and F4/80 (macrophage marker). (h, i) The dynamic changes in glycolysis and TCA cycle were measured by ECAR and OCR, respectively, using a Seahorse extracellular flux analyzer from different treatments (*n* = 4). (j) The level of lactic acid in different treatments (*n* = 3). Data are presented as mean ± SD. Statistical comparisons were performed using the Mann–Whitney test and unpaired *t*‐test. *p < 0.05, **p < 0.01, ***p < 0.001, ****p < 0.0001. DSS, dextran sulfate sodium; ECAR, extracellular acidification rate; EcN‐T, *Escherichia coli* Nissle 1917‐tyrosinase; OCR, oxygen consumption rate; TCA, tricarboxylic acid; KEGG, kyoto encyclopedia of genes and genomes; q‐PCR, quantitative polymerase chain reaction; SD, standard deviation.

We further performed transcriptomic analysis on intestinal tissues of mice under different treatments. Venn diagram analysis revealed significant transcriptomic differences among the healthy group, DSS‐induced colitis model group, and EcN‐T treatment group (Figure S8). Further analysis of the volcano plot comparing gene expression between the DSS‐induced colitis model group and the EcN‐T treatment group demonstrated substantial differences, especially the glycolysis‐related genes HIF‐1α, GLUT1, and PFKFB3, which were significantly downregulated after EcN‐T treatment (Figure 6a). Gene ontology analysis revealed significant differences in several biological processes among the differently treated mice, including cell adhesion molecule binding, chemokine‐mediated signaling pathway, glycolytic process, bicellular tight junction, tricarboxylic acid cycle, vascular endothelial growth factor receptor signaling pathway, gluconeogenesis, receptor signaling pathway via Janus Kinase–Signal Transducer and Activator of Transcription (JAK–STAT), TNF‐mediated signaling pathway, and apoptotic process(Figure 6b). Additionally, Kyoto Encyclopedia of Genes and Genomes (KEGG) analysis was performed to further explore related signaling pathways (Figure 6c). The analysis showed that the differentially expressed genes between the DSS‐induced colitis model group and the EcN‐T treatment group were enriched in 10 key pathways, including cell adhesion molecules, TNF signaling pathway, IBD, HIF‐1 signaling pathway, Nuclear Factor kappa‐light‐chain‐enhancer of Activated B Cells (NF‐kB) signaling pathway, tight junction, glycolysis/gluconeogenesis, Toll‐like receptor signaling pathway, AMP‐activated Protein Kinase (AMPK) signaling pathway, and Mechanistic Target of Rapamycin (mTOR) signaling pathway. To validate the differential gene expression in colonic tissues of mice from different treatment groups, we performed Western blot and q‐PCR analyses(Figure 6d,e). The results showed that, compared to the DSS‐induced colitis model group, EcN‐T treatment significantly downregulated the expression of HIF‐1α, GLUT1, and PFKFB3, which was consistent with the results of the volcano plot analysis.

To further identify the primary cell type involved, we performed immune infiltration analysis on transcriptomic data from mouse colonic tissues using CIBERSORT.^44^, ^45^, ^46^ The results showed a significant reduction in M1 macrophages in the EcN‐T treatment group compared to the DSS‐induced colitis model group (Figure 6f). Immunofluorescence staining (F4/80 + CD86 double staining) of mouse colonic tissues further confirmed that EcN‐T treatment markedly reduced the number of M1 macrophages (Figure 6g). To further validate the regulatory effect of EcN‐T on M1 macrophage polarization, we co‐cultured EcN‐T with RAW264.7 cells using the Transwell method. By measuring ECAR and OCR, we assessed the dynamic changes in glycolysis and the tricarboxylic acid (TCA) cycle, respectively. The results showed that LPS stimulation significantly increased ECAR and OCR in macrophages polarized to the M1 phenotype, consistent with previous studies, while EcN‐T treatment significantly reversed this phenomenon (Figure 6h,i).^47^ In addition, we measured lactate production, a key product of glycolysis, and found that lactate levels were significantly increased in macrophages under LPS stimulation, while EcN‐T treatment significantly reduced lactate production (Figure 6j). ELISA analysis of inflammatory cytokines in cell culture supernatants revealed that the levels of pro‐inflammatory cytokines IL‐1b, TNF‐α, and IL‐6 were significantly reduced in the EcN‐T co‐culture group, while the level of the anti‐inflammatory cytokine IL‐10 was significantly increased (Figure S9). Further flow cytometry analysis showed that LPS stimulation promoted polarization of M0 macrophages into the M1 phenotype, as indicated by CD86 staining, while co‐culture with EcN‐T significantly inhibited this polarization process (Figure S10). Finally, we used Western blot and q‐PCR on the RAW264.7 cell line to analyze the expression of HIF‐1α, GLUT1, and PFKFB3. The results showed that EcN‐T treatment significantly downregulated the expression levels of these genes, consistent with the volcano plot and other experimental results (Figures S11 and S12).

Through comprehensive analysis, we speculate that EcN‐T exerts its significant anti‐inflammatory effects by modulating HIF‐1α‐dependent glycolytic reprogramming in macrophages, effectively suppressing M1 macrophage polarization.

### 3.7 Biosafety evaluation of EcN‐T

Lastly, we evaluated the toxicity of EcN‐T in animals. EcN‐T was administered orally to healthy mice at therapeutic doses every 2 days, for a total of four times. On Day 10, mice were sacrificed and serum was collected for blood routine and biochemical tests, as well as major organs (heart, liver, spleen, lung, kidney). As expected, the treatment did not alter the levels of red blood cells, white blood cells, hemoglobin, platelets, lymphocytes, and neutrophils; these parameters were consistent with those of healthy control mice (Figure S13). Liver and kidney function parameters such as aspartate aminotransferase, alanine aminotransferase, blood urea nitrogen, and creatinine were all within the normal range, indicating no hepatic or renal toxicity (Figure S14). Histological analysis of major organs from EcN‐T‐treated mice showed no significant differences compared to healthy mice, indicating no apparent toxicity or pathological changes (Figure S15). Overall, these results suggest that EcN‐T is well‐tolerated in treated mice and does not induce adverse side effects, supporting its biosafety for potential therapeutic applications.

## 4 DISCUSSION

Our study demonstrates that the genetically engineered probiotic EcN‐T effectively overexpresses tyrosinase to biosynthesize melanin@EcN‐T, exhibiting excellent biocompatibility, antioxidant, and anti‐inflammatory properties. We observed that melanin@EcN‐T nanoparticles could efficiently scavenge ROS and show high biodegradability in an H₂O₂‐rich environment. In vitro experiments indicated that melanin@EcN‐T significantly reduced ROS levels and improved cell survival in H₂O₂‐induced RAW264.7 cell inflammation models.

DSS‐induced colitis model was used for this study because it is easy to establish and suitable for investigating inflammation recovery in the gastrointestinal tract.^48^ Our research found that oral administration of EcN‐T effectively upregulated the expression of tight junction‐related proteins in colon tissues, efficiently scavenged ROS, and modulated inflammatory responses, leading to a significant reduction in pro‐inflammatory cytokines and an increase in anti‐inflammatory cytokines. More importantly, the EcN‐T treatment group showed a marked alleviation of DSS‐induced colitis symptoms, including rapid weight recovery, reduced colonic mucosal damage, and increased colonic SOD activity, whereas the groups treated with either EcN or melanin alone did not show protective effects against DSS‐induced colitis.

The gut microbiota dysbiosis plays a crucial role in the pathogenesis of UC. These microbial communities are involved not only in digestion and nutrient absorption but also in immune system regulation. Studies have shown that patients with UC have significantly reduced gut microbiota diversity, which may lead to impaired gut barrier function and an overreactive immune system. Modulating the gut microbiota, such as through probiotic therapy or fecal transplantation, holds promise for improving UC symptoms and restoring gut ecological balance. Therefore, understanding and regulating the gut microbiota is a critical direction for UC treatment.^49^, ^50^ Our study found that the EcN‐T treatment group could regulate the gut microbiota and achieve therapeutic effects by increasing the abundance of beneficial bacteria, such as *Bacteroides* and *Lactobacillus*. Research indicates that SCFAs have significant anti‐inflammatory functions and the ability to regulate the immune system, playing an important role in the prevention and treatment of UC.^51^ Our study found that the EcN‐T treatment group significantly increased the levels of SCFAs such as butyric acid, valeric acid, and propionic acid. Butyrate acid plays a key role in maintaining colonic homeostasis, serving as an important energy source for colonic epithelial cells, inhibiting the release of inflammatory cytokines, and upregulating the expression of tight junction proteins to enhance epithelial barrier integrity.^38^, ^48^ Additionally, reports indicate that butyrate can achieve therapeutic effects by inhibiting M1 macrophage polarization.^52^

Macrophages are key effector cells in the innate immune system and play an important role in gut health and the development of UC. They are abundant in tissues, particularly in the gut, where they regulate gut homeostasis and repair through different phenotypes (such as M1 and M2). The polarization and balance of macrophages are crucial, and their anti‐inflammatory properties and polarization balance are particularly important for UC treatment.^53^ Our transcriptomic analysis showed that EcN‐T treatment significantly reduced M1 macrophage polarization. KEGG enrichment and differential gene analyses revealed that EcN‐T mainly affected glycolysis, which was confirmed by measuring glycolysis indicators (ECAR and OCR). Thus, we concluded that EcN‐T regulates macrophage polarization via HIF‐1α‐dependent glycolytic reprogramming, suppressing M1 polarization and lowering pro‐inflammatory cytokine expression. This finding offers a new perspective on UC treatment.

Despite the potential of EcN‐T in treating UC demonstrated by our study, there are some limitations. First, gastric acid and bile salts in the gastrointestinal tract can lead to the inactivation of probiotics, thereby reducing their therapeutic efficacy. Second, larger‐scale experiments in mice and other UC animal models are needed to further confirm the therapeutic effects and mechanisms of engineered probiotics EcN‐T. Third, although we explored the therapeutic mechanism and reviewed relevant literature, we did not use small molecule inhibitors or other methods to further verify the logical correctness of the pathway's upstream and downstream components. It should also be noted that only male mice were utilized in the present study, by which the generalizability of the findings to female subjects may be limited. In future investigations, both male and female mice should be incorporated so that sex‐related differences in the mechanisms and outcomes under investigation can be comprehensively assessed.

## 5 CONCLUSIONS

In summary, this study highlights the potential application of the engineered probiotic EcN‐T in the treatment of UC. Through genetic modification, EcN‐T effectively overexpresses tyrosinase to biosynthesize melanin, showing excellent biocompatibility, antioxidant, and anti‐inflammatory properties. These properties aid in scavenging ROS and restoring the intestinal mucosal barrier. Additionally, they promote the growth of beneficial bacteria while reducing harmful bacteria, thereby restoring the balance of the gut microbiota. At the same time, EcN‐T increases levels of SCFAs. These combined effects are crucial for maintaining colonic homeostasis and exerting anti‐inflammatory effects. Moreover, the study found that EcN‐T can inhibit M1 macrophage polarization through HIF‐1α‐dependent glycolytic reprogramming, demonstrating immune regulatory capabilities. Overall, these findings provide significant insights into the therapeutic application of EcN‐T, suggesting a promising approach for UC treatment by integrating strategies of gut microbiota modulation and immune regulation.

## CONFLICT OF INTEREST STATEMENT

The authors declare no conflicts of interest.

## Supporting information


**Data S1.** Supporting Information.

## Data Availability

The 16S rRNA gene sequencing raw sequence reads (fastq) and RNA‐seq sequencing data (fastq) produced in this study are available in the GSA Sequence Read Archive under accession numbers CRA021177 and CRA021167, respectively. The targeted metabolomic data are available in the OMIX database under OMIX008244. All other data are contained within the main manuscript and supplemental files.

## References

[1] Le Berre C , Honap S , Peyrin‐Biroulet L . Ulcerative colitis. Lancet. 2023;402(10401):571‐584. 37573077 10.1016/S0140-6736(23)00966-2

[2] Kobayashi T , Siegmund B , Le Berre C , et al. Ulcerative colitis. Nat Rev Dis Primers. 2020;6(1):74. 32913180 10.1038/s41572-020-0205-x

[3] Zhang C , Wang H , Yang X , et al. Oral zero‐valent‐molybdenum nanodots for inflammatory bowel disease therapy. Sci Adv. 2022;8(37):eabp9882. 36112678 10.1126/sciadv.abp9882 PMC9481133

[4] Fu W , Huang Z , Li W , et al. Copper‐luteolin nanocomplexes for mediating multifaceted regulation of oxidative stress, intestinal barrier, and gut microbiota in inflammatory bowel disease. Bioact Mater. 2025;46:118‐133. 39760067 10.1016/j.bioactmat.2024.12.004 PMC11697280

[5] Zhang X , Yang H , He Y , et al. Yeast‐inspired orally‐administered nanocomposite scavenges oxidative stress and restores gut immune homeostasis for inflammatory bowel disease treatment. ACS Nano. 2025;19(7):7350‐7369. 39943645 10.1021/acsnano.4c18099

[6] Ferrante M , Sabino J . Efficacy of JAK inhibitors in ulcerative colitis. J Crohns Colitis. 2020;14(Supplement_2):S737‐S745. 31879750 10.1093/ecco-jcc/jjz202 PMC7395310

[7] Feuerstein JD , Moss AC , Farraye FA . Ulcerative colitis. Mayo Clin Proc. 2019;94(7):1357‐1373. 31272578 10.1016/j.mayocp.2019.01.018

[8] Huang L , Hu W , Huang LQ , et al. “Two‐birds‐one‐stone” oral nanotherapeutic designed to target intestinal integrins and regulate redox homeostasis for UC treatment. Sci Adv. 2024;10(30):eado7438. 39047093 10.1126/sciadv.ado7438 PMC11268407

[9] Praveschotinunt P , Duraj‐Thatte AM , Gelfat I , Bahl F , Chou DB , Joshi NS . Engineered *E. coli* Nissle 1917 for the delivery of matrix‐tethered therapeutic domains to the gut. Nat Commun. 2019;10(1):5580. 31811125 10.1038/s41467-019-13336-6 PMC6898321

[10] Liu Y , Gao C , Li G , et al. Melanin nanoparticle‐modified probiotics for targeted synergistic therapy of ulcerative colitis. ACS Appl Mater Interfaces. 2024;16(25):31950‐31965. 38861025 10.1021/acsami.4c02914

[11] Lynch JP , Goers L , Lesser CF . Emerging strategies for engineering *Escherichia coli* Nissle 1917‐based therapeutics. Trends Pharmacol Sci. 2022;43(9):772‐786. 35232591 10.1016/j.tips.2022.02.002 PMC9378478

[12] Redenti A , Im J , Redenti B , et al. Probiotic neoantigen delivery vectors for precision cancer immunotherapy. Nature. 2024;635:453‐461. 39415001 10.1038/s41586-024-08033-4 PMC11560847

[13] Chen H , Lei P , Ji H , et al. Advances in *Escherichia coli* Nissle 1917 as a customizable drug delivery system for disease treatment and diagnosis strategies. Mater Today Bio. 2023;18:100543. 10.1016/j.mtbio.2023.100543 PMC9840185 36647536

[14] Zhao Z , Xu S , Zhang W , Wu D , Yang G . Probiotic *Escherichia coli* Nissle 1917 for inflammatory bowel disease applications. Food Funct. 2022;13(11):5914‐5924. 35583304 10.1039/d2fo00226d

[15] Huang Q , Yang Y , Zhu Y , et al. Oral metal‐free melanin nanozymes for natural and durable targeted treatment of inflammatory bowel disease (IBD). Small. 2023;19(19):e2207350. 36760016 10.1002/smll.202207350

[16] Chen Y , Liu H , Huang H , et al. Squid ink polysaccharides protect human fibroblast against oxidative stress by regulating NADPH oxidase and connexin43. Front Pharmacol. 2020;10:1574. 32009967 10.3389/fphar.2019.01574 PMC6978904

[17] Han M , Zhao Y , Song W , Wang C , Mu C , Li R . Changes in microRNAs expression profile of mimetic aging mice treated with melanin from *Sepiella japonica* ink. J Agric Food Chem. 2020;68(20):5616‐5622. 32345009 10.1021/acs.jafc.0c00291

[18] Kunwar A , Adhikary B , Jayakumar S , et al. Melanin, a promising radioprotector: mechanisms of actions in a mice model. Toxicol Appl Pharmacol. 2012;264(2):202‐211. 22968190 10.1016/j.taap.2012.08.002

[19] Xie J , Liu L , Li H , Che H , Xie W . Ink melanin from Sepiapharaonis ameliorates colitis in mice via reducing oxidative stress, andprotecting the intestinal mucosal barrier. Food Res Int. 2022;151:110888. 34980415 10.1016/j.foodres.2021.110888

[20] El‐Naggar NE‐A , El‐Ewasy SM . Bioproduction, characterization, anticancer and antioxidant activities of extracellular melanin pigment produced by newly isolated microbial cell factories Streptomyces glaucescens NEAE‐H. Sci Rep. 2017;7(1):42129. 28195138 10.1038/srep42129 PMC5307326

[21] Fu M , Yang Y , Zhang Z , et al. Biosynthesis of melanin nanoparticles for photoacoustic imaging guided photothermal therapy. Small. 2022;19(14):2205343. 10.1002/smll.202205343 36581563

[22] Zhang X , Yuan Z , Wu J , et al. An orally‐administered nanotherapeutics with carbon monoxide supplying for inflammatory bowel disease therapy by scavenging oxidative stress and restoring gut immune homeostasis. ACS Nano. 2023;17(21):21116‐21133. 37843108 10.1021/acsnano.3c04819

[23] Han X , Guo J , You Y , et al. A fast and accurate way to determine short chain fatty acids in mouse feces based on GC‐MS. J Chromatogr B Analyt Technol Biomed Life Sci. 2018;1099:73‐82. 10.1016/j.jchromb.2018.09.013 30243116

[24] Hsu YL , Chen CC , Lin YT , et al. Evaluation and optimization of sample handling methods for quantification of short‐chain fatty acids in human fecal samples by GC‐MS. J Proteome Res. 2019;18(5):1948‐1957. 30895795 10.1021/acs.jproteome.8b00536

[25] Meng Q , Guo J , Lv K , et al. 5S‐Heudelotinone alleviates experimental colitis by shaping the immune system and enhancing the intestinal barrier in a gut microbiota‐dependent manner. Acta Pharm Sin B. 2024;14(5):2153‐2176. 38799623 10.1016/j.apsb.2024.02.020 PMC11120280

[26] Duan Y , Xiong D , Wang Y , Li H , Dong H , Zhang J . Toxic effects of ammonia and thermal stress on the intestinal microbiota and transcriptomic and metabolomic responses of *Litopenaeus vannamei* . Sci Total Environ. 2021;754:141867. 32898779 10.1016/j.scitotenv.2020.141867

[27] Chen Y , Zhou Y , Ren R , Chen Y , Lei J , Li Y . Harnessing lipid metabolism modulation for improved immunotherapy outcomes in lung adenocarcinoma. J Immunother Cancer. 2024;12(7):e008811. 38977328 10.1136/jitc-2024-008811 PMC11256034

[28] Wang X , Kinziabulatova L , Bortoli M , et al. Indole‐5,6‐quinones display hallmark properties of eumelanin. Nat Chem. 2023;15(6):787‐793. 37037912 10.1038/s41557-023-01175-4

[29] Sun D , Liu K , Li Y , et al. Intrinsically bioactive manganese‐eumelanin nanocomposites mediated antioxidation and anti‐neuroinflammation for targeted theranostics of traumatic brain injury. Adv Healthc Mater. 2022;11(16):e2200517. 35695187 10.1002/adhm.202200517

[30] Wakamatsu K , Ito S . Recent advances in characterization of melanin pigments in biological samples. Int J Mol Sci. 2023;24(9):8305. 37176019 10.3390/ijms24098305 PMC10179066

[31] He L , He T , Farrar S , Ji L , Liu T , Ma X . Antioxidants maintain cellular redox homeostasis by elimination of reactive oxygen species. Cell Physiol Biochem. 2017;44(2):532‐553. 29145191 10.1159/000485089

[32] Tsikas D . Assessment of lipid peroxidation by measuring malondialdehyde (MDA) and relatives in biological samples: analytical and biological challenges. Anal Biochem. 2017;524:13‐30. 27789233 10.1016/j.ab.2016.10.021

[33] Breugelmans T , Oosterlinck B , Arras W , et al. The role of mucins in gastrointestinal barrier function during health and disease. Lancet Gastroenterol Hepatol. 2022;7(5):455‐471. 35397245 10.1016/S2468-1253(21)00431-3

[34] Tsukita S , Yamazaki Y , Katsuno T , Tamura A , Tsukita S . Tight junction‐based epithelial microenvironment and cell proliferation. Oncogene. 2008;27(55):6930‐6938. 19029935 10.1038/onc.2008.344

[35] Niessen CM . Tight junctions/adherens junctions: basic structure and function. J Invest Dermatol. 2007;127(11):2525‐2532. 17934504 10.1038/sj.jid.5700865

[36] Vrancken G , Gregory AC , Huys GRB , Faust K , Raes J . Synthetic ecology of the human gut microbiota. Nat Rev Microbiol. 2019;17(12):754‐763. 31578461 10.1038/s41579-019-0264-8

[37] Deleu S , Machiels K , Raes J , Verbeke K , Vermeire S . Short chain fatty acids and its producing organisms: an overlooked therapy for IBD? EBioMedicine. 2021;66:103293. 33813134 10.1016/j.ebiom.2021.103293 PMC8047503

[38] Ruan G , Chen M , Chen L , et al. Roseburia intestinalis and its metabolite butyrate inhibit colitis and upregulate TLR5 through the SP3 signaling pathway. Nutrients. 2022;14(15):3041. 35893896 10.3390/nu14153041 PMC9332583

[39] El Hage R , Hernandez‐Sanabria E , Van de Wiele T . Emerging trends in “smart probiotics”: functional consideration for the development of novel health and industrial applications. Front Microbiol. 2017;8:1889. 29033923 10.3389/fmicb.2017.01889 PMC5626839

[40] Hiippala K , Kainulainen V , Kalliomaki M , Arkkila P , Satokari R . Mucosal prevalence and interactions with the epithelium indicate commensalism of *Sutterella* spp. Front Microbiol. 2016;7:1706. 27833600 10.3389/fmicb.2016.01706 PMC5080374

[41] Li C , Peng K , Xiao S , Long Y , Yu Q . The role of lactobacillus in inflammatory bowel disease: from actualities to prospects. Cell Death Discov. 2023;9(1):361. 37773196 10.1038/s41420-023-01666-w PMC10541886

[42] Brown EM , Ke X , Hitchcock D , et al. Bacteroides‐derived sphingolipids are critical for maintaining intestinal homeostasis and Symbiosis. Cell Host Microbe. 2019;25(5):668‐680. 31071294 10.1016/j.chom.2019.04.002 PMC6544385

[43] Zafar H , Saier MH Jr . Gut Bacteroides species in health and disease. Gut Microbes. 2021;13(1):1‐20. 10.1080/19490976.2020.1848158 PMC7872030 33535896

[44] Chen Z , Huang A , Sun J , Jiang T , Qin FX , Wu A . Inference of immune cell composition on the expression profiles of mouse tissue. Sci Rep. 2017;7:40508. 28084418 10.1038/srep40508 PMC5233994

[45] Newman AM , Liu CL , Green MR , et al. Robust enumeration of cell subsets from tissue expression profiles. Nat Methods. 2015;12(5):453‐457. 25822800 10.1038/nmeth.3337 PMC4739640

[46] Petitprez F , Levy S , Sun CM , et al. The murine microenvironment cell population counter method to estimate abundance of tissue‐infiltrating immune and stromal cell populations in murine samples using gene expression. Genome Med. 2020;12(1):86. 33023656 10.1186/s13073-020-00783-w PMC7541325

[47] Liu J , Cao X . Glucose metabolism of TAMs in tumor chemoresistance and metastasis. Trends Cell Biol. 2023;33(11):967‐978. 37080816 10.1016/j.tcb.2023.03.008

[48] Zhou J , Li M , Chen Q , et al. Programmable probiotics modulate inflammation and gut microbiota for inflammatory bowel disease treatment after effective oral delivery. Nat Commun. 2022;13(1):3432. 35701435 10.1038/s41467-022-31171-0 PMC9198027

[49] Kostic AD , Xavier RJ , Gevers D . The microbiome in inflammatory bowel disease: current status and the future ahead. Gastroenterology. 2014;146(6):1489‐1499. 24560869 10.1053/j.gastro.2014.02.009 PMC4034132

[50] Paramsothy S , Kamm MA , Kaakoush NO , et al. Multidonor intensive faecal microbiota transplantation for active ulcerative colitis: a randomised placebo‐controlled trial. Lancet. 2017;389(10075):1218‐1228. 28214091 10.1016/S0140-6736(17)30182-4

[51] Zhang Z , Zhang H , Chen T , Shi L , Wang D , Tang D . Regulatory role of short‐chain fatty acids in inflammatory bowel disease. Cell Commun Signal. 2022;20(1):64. 35546404 10.1186/s12964-022-00869-5 PMC9097439

[52] Zhao S , Zhang H , Zhu H , et al. Gut microbiota promotes macrophage M1 polarization in hepatic sinusoidal obstruction syndrome via regulating intestinal barrier function mediated by butyrate. Gut Microbes. 2024;16(1):2377567. 39012957 10.1080/19490976.2024.2377567 PMC11253885

[53] Zhang K , Guo J , Yan W , Xu L . Macrophage polarization in inflammatory bowel disease. Cell Commun Signal. 2023;21(1):367. 38129886 10.1186/s12964-023-01386-9 PMC10734116

