# Supplementary material for: Engineered probiotic alleviates ulcerative colitis by inhibiting M1 macrophage polarization via glycolytic reprogramming
Source: Bioeng Transl Med. 2025 Aug 29;10(6):e70067. doi: 10.1002/btm2.70067 (PMC12617548; doi:10.1002/btm2.70067)
Supplement: Supplementary file 1 — Data S1. Supporting Information. [file BTM2-10-e70067-s001.docx]

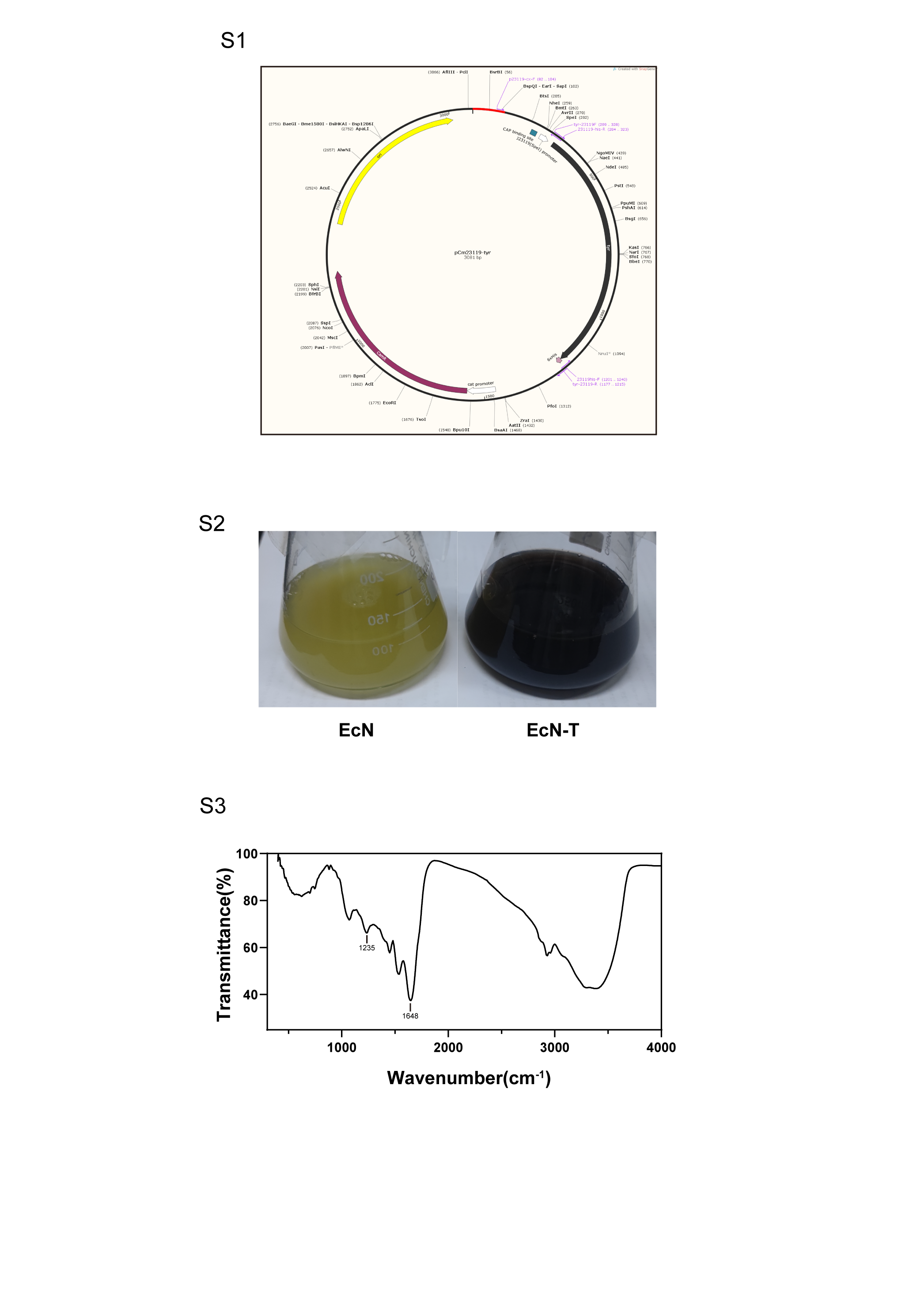
Supplement Figure 1: Plasmid atlas of pCm23119-tyr-6×His which carried tyrosinase gene Tyr1 derived from bacillus megaterium.


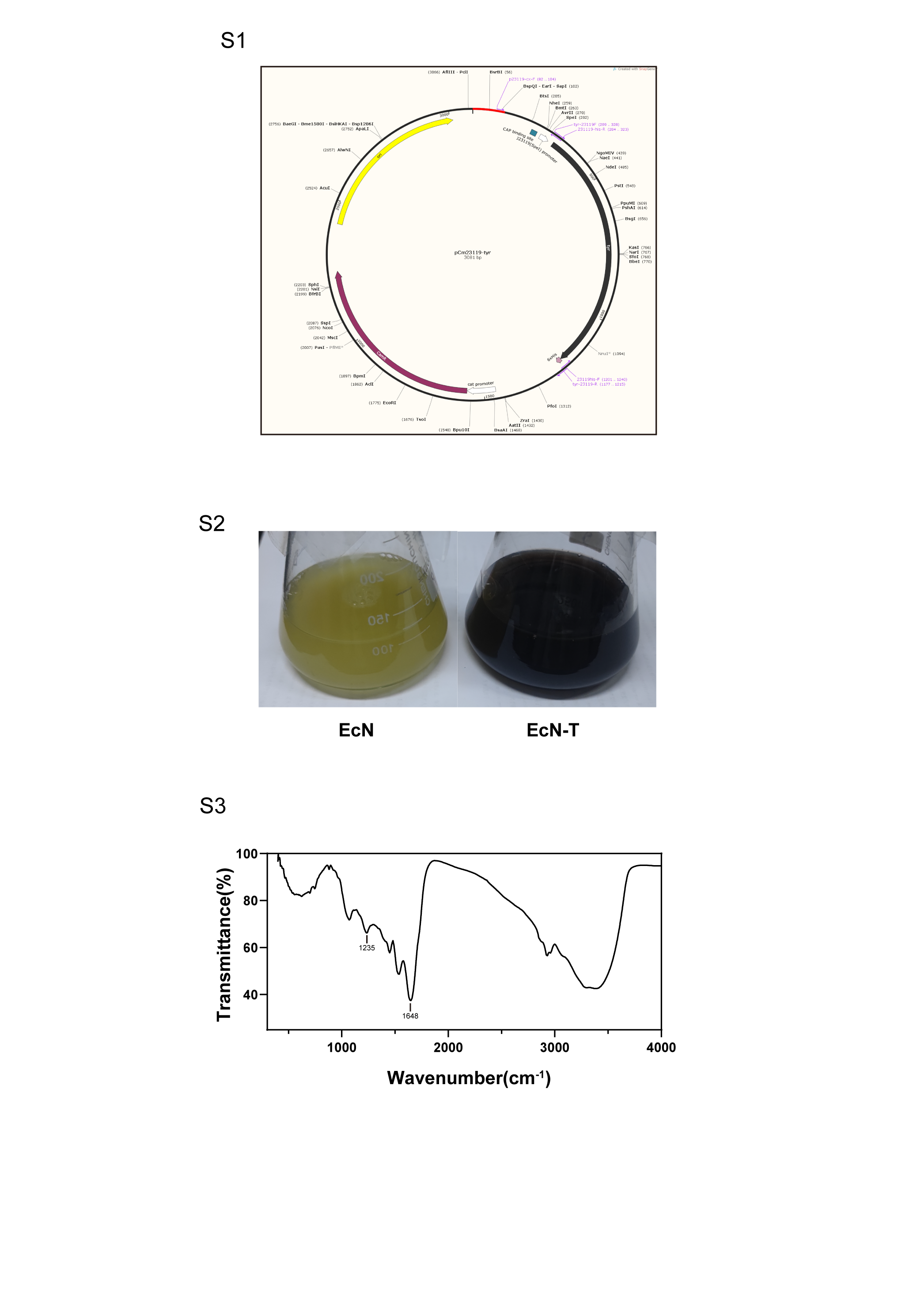


Supplement Figure 2: Image of EcN and EcN-T.


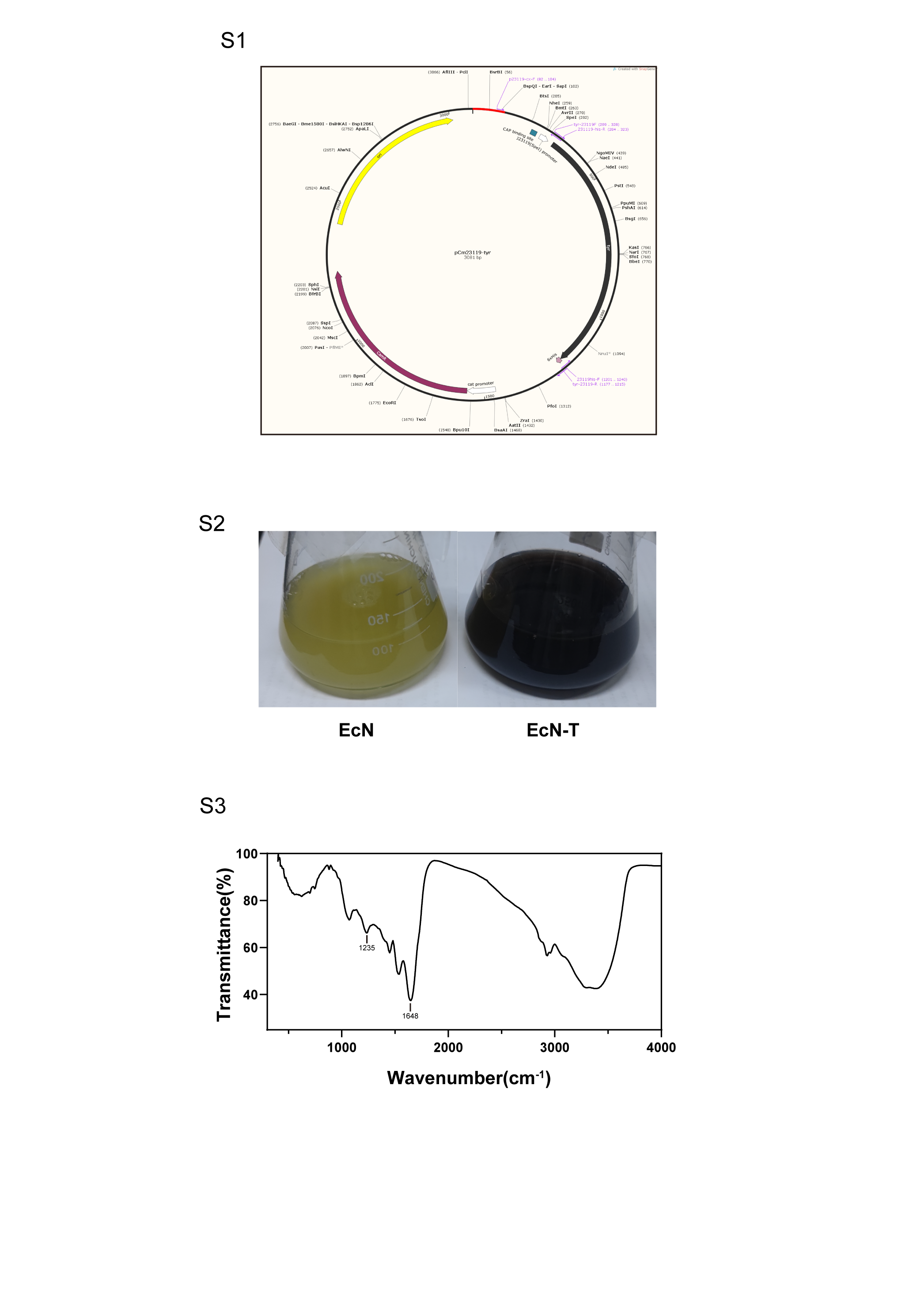


Supplement Figure 3: Fourier transform infrared spectra of product melanin@EcN-T.


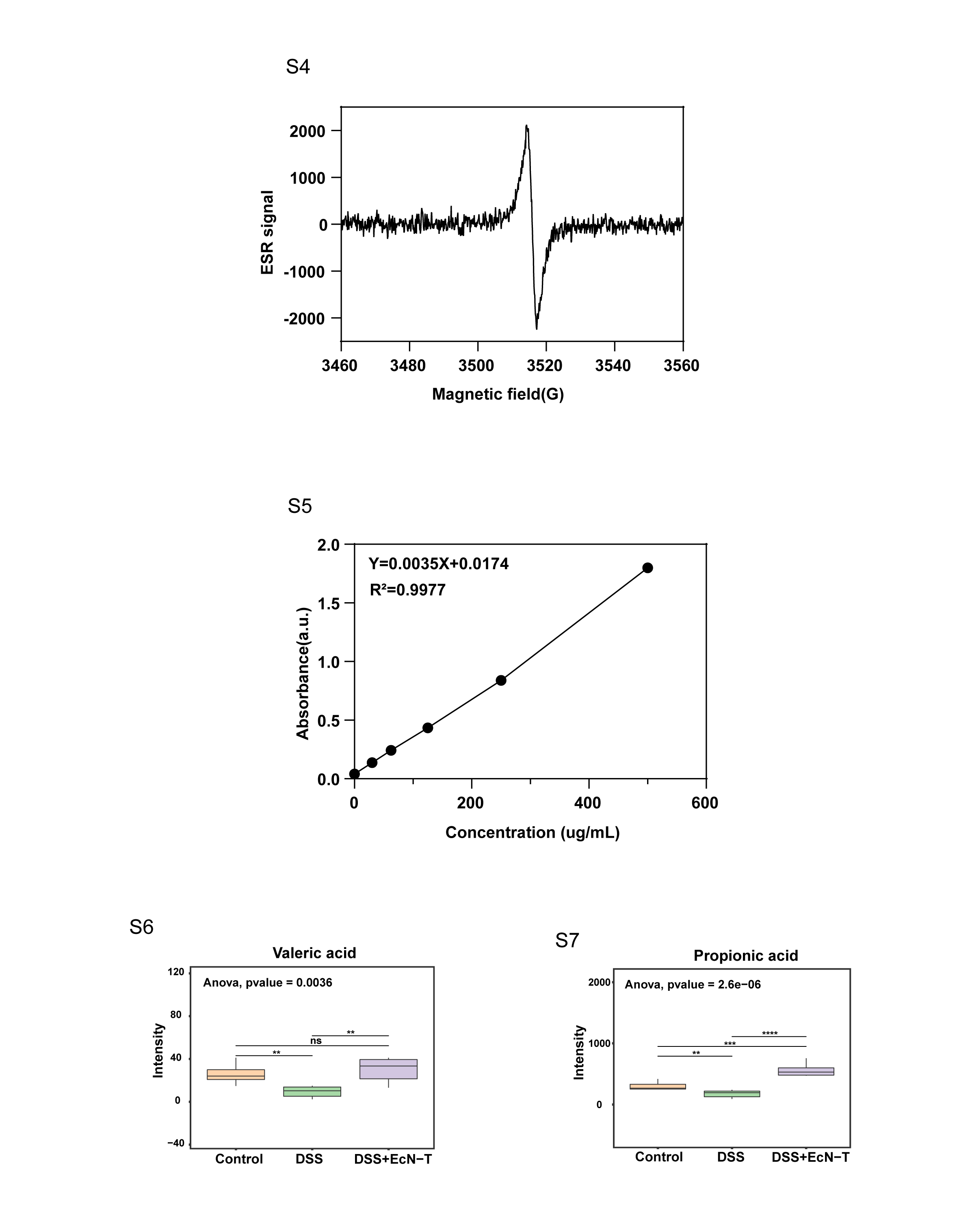


Supplement Figure 4: ESR spectra of melanin@EcN-T.


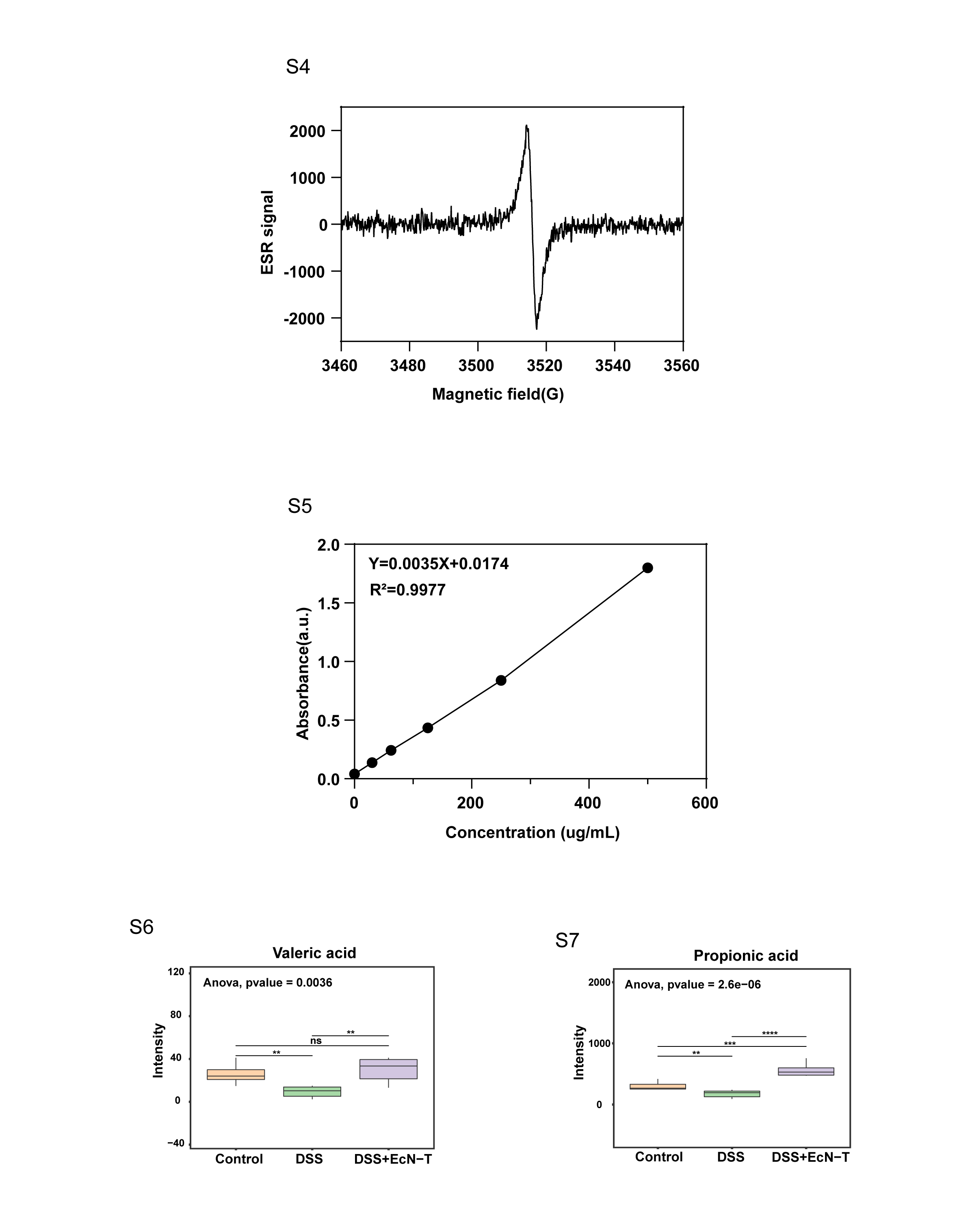


Supplement Figure 5: The concentration-dependent absorbance of melanin@EcN-T in the UV-Vis-NIR region at the wavelengths 492 nm.


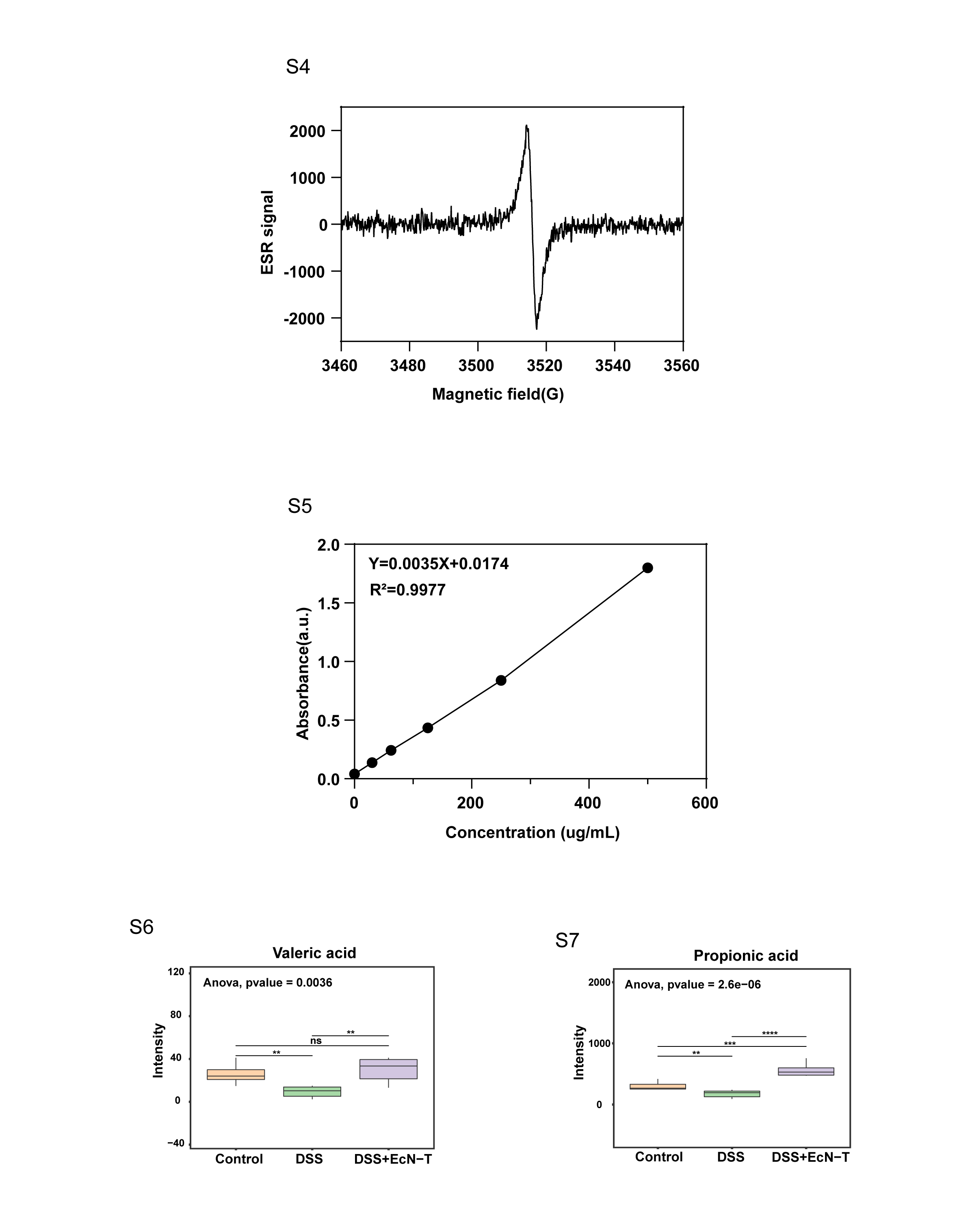


Supplement Figure 6-7: Quantitative box plot of Valeric and Propionic expression levels in different treatments (n=6).

Supplement Figure 8: Venn diagram of whole-transcriptome RNA-seq analysis showing differentially expressed genes in pairs between Control, DSS and DSS+EcN-T group (p < 0.05 and fold difference log2FC) > 2).

Supplement Figure 9: Expression of inflammatory cytokines in the supernatant of RAW264.7 cells under different treatments (n=4).

Supplement Figure 10: The expression of CD86 in different treatment group using Flow Cytometry.

Supplement Figure 11: The Western blot of HIF-1a, PFKFB3, and GLUT1 from different treatments (n=3).


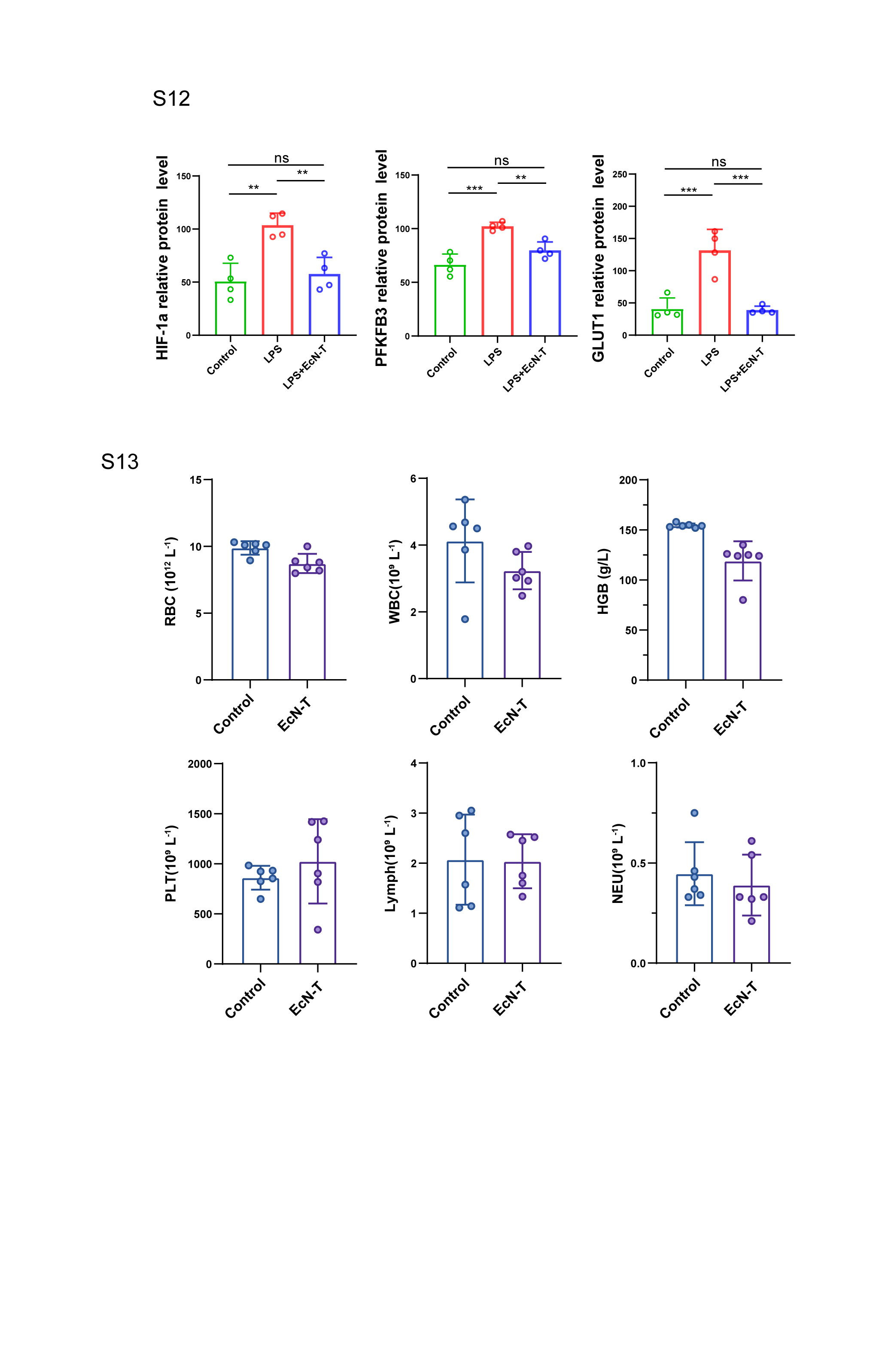


Supplement Figure 12: The western blot quantification results of HIF-1a, PFKFB3, and GLUT1 from different treatments (n=4).


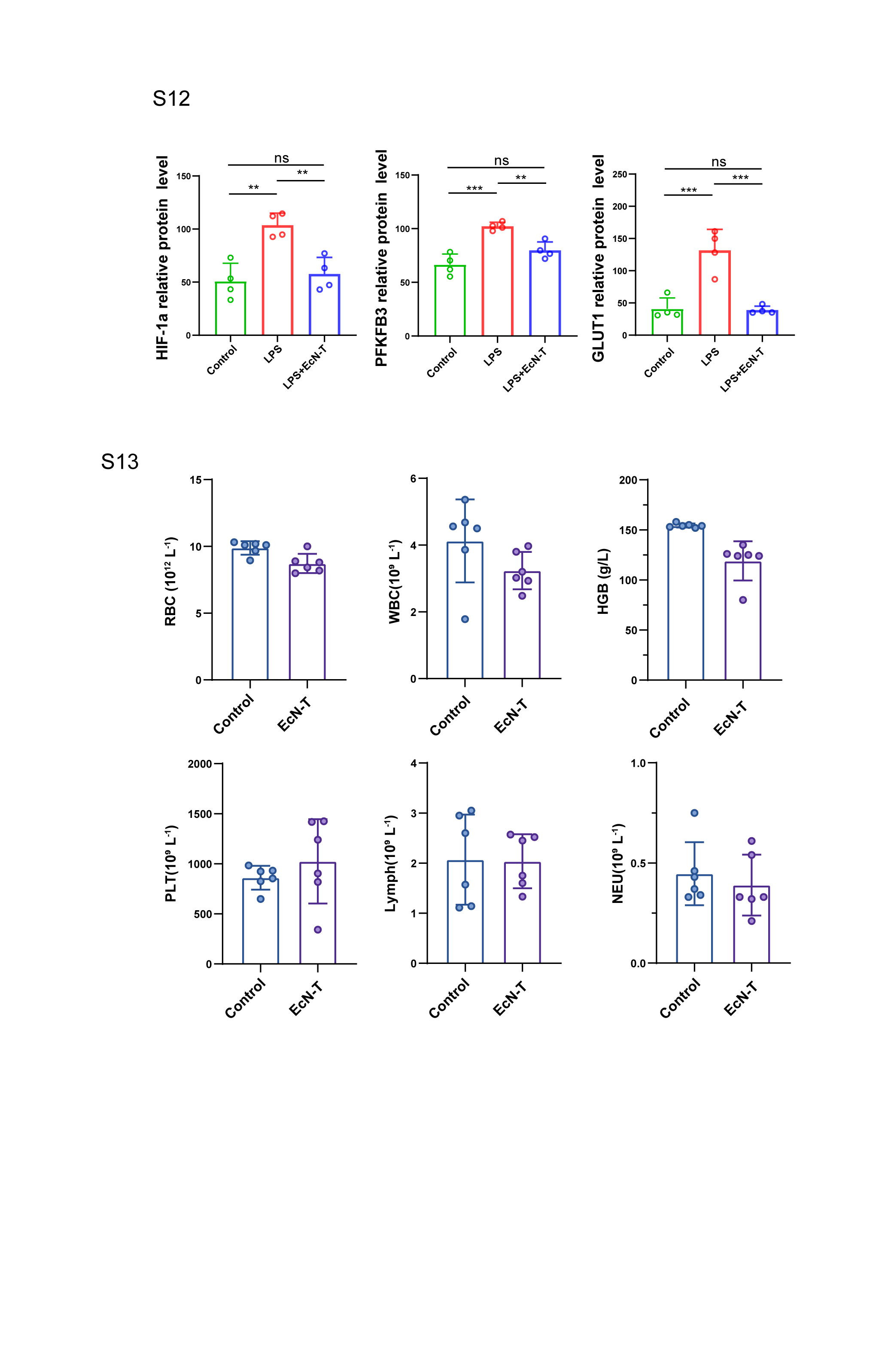


Supplement Figure 13: Hemanalysis of mice After gavage of EcN-T at 1x10^8^ CFU, compared with that injected with saline. Including：RBC: red blood cell, WBC: white blood cell, PLT: platelet, Lymph: lymphocyte, NEU:neutrophils (n=6).


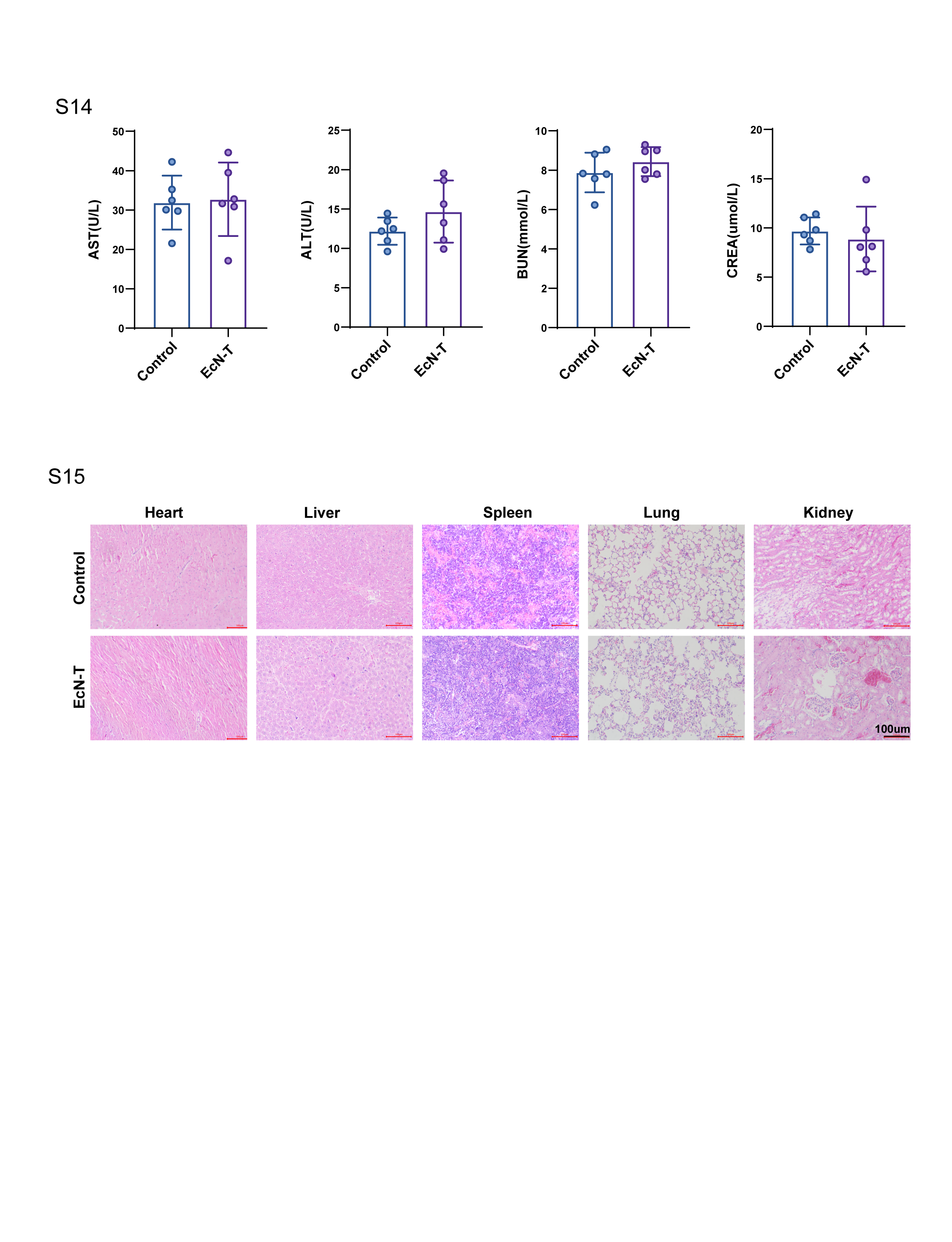


Supplement Figure 14: Blood biochemistry examination of AST, ALT, BUN, CREA in mice after different treatments. (n=6).


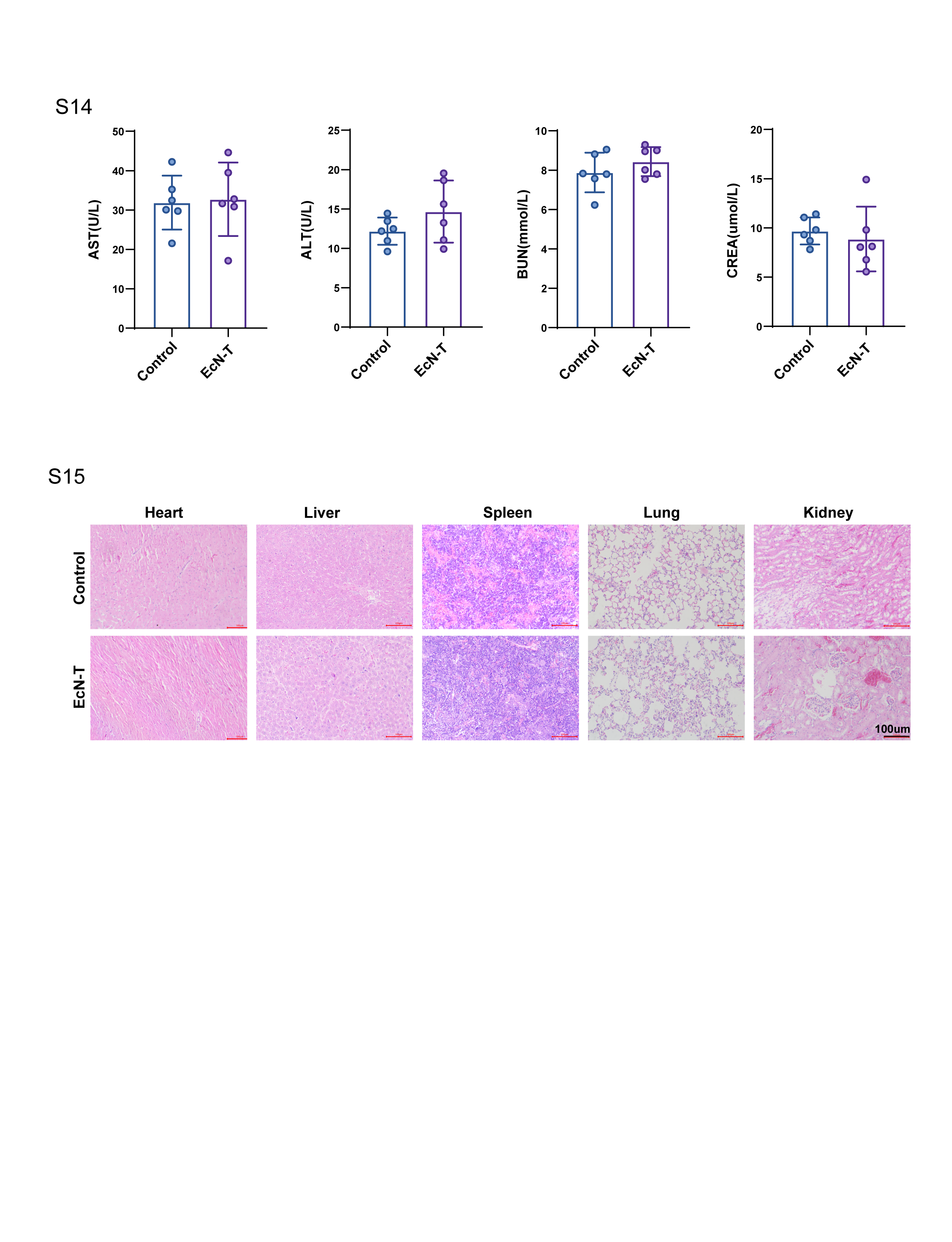


Supplement Figure 15: H&E staining of major organs of mice after the different treatments. Scale bar: 100um.

Supplementary text 1

Nucleotide sequencec for Tyr:

AtgGGTAATAAGTACCGTGTTCGTAAAAATGTGCTGCATCTGACCGATACCGAAAAACGCGATTTTGTTCGTACCGTTCTGATTCTGAAAGAAAAAGGCATCTATGATCGTTATATCGCATGGCATGGCGCAGCCGGCAAATTTCATACCCCGCCGGGTAGTGATCGCAATGCCGCCCATATGAGTAGTGCATTTCTGCCGTGGCATCGTGAATATCTGCTGCGTTTTGAACGTGATCTGCAGAGTATTAATCCGGAAGTGACCCTGCCGTATTGGGAATGGGAAACCGATGCCCAGATGCAGGACCCTAGTCAGAGCCAGATTTGGAGTGCAGATTTTATGGGTGGTAATGGCAATCCGATTAAGGATTTTATTGTGGATACCGGCCCGTTTGCAGCCGGTCGCTGGACCACCATTGATGAACAGGGTAATCCGAGTGGCGGCCTGAAACGCAATTTTGGCGCCACCAAAGAAGCCCCGACCCTGCCGACCCGTGATGATGTTCTGAATGCACTGAAAATTACCCAGTATGATACCCCGCCGTGGGATATGACCAGTCAGAATAGTTTTCGTAATCAGCTGGAAGGTTTTATTAATGGCCCGCAGCTGCATAATCGTGTGCATCGCTGGGTGGGCGGTCAGATGGGCGTTGTTCCGACCGCCCCGAATGATCCGGTTTTCTTTCTGCATCATGCAAATGTGGATCGTATTTGGGCCGTGTGGCAGATTATTCATCGCAATCAGAATTATCAGCCGATGAAAAATGGCCCGTTTGGTCAGAATTTTCGCGATCCGATGTATCCGTGGAATACCACCCCGGAAGATGTGATGAATCATCGTAAACTGGGTTATGTTTATGATATTGAACTGCGTAAAAGCAAACGCAGTAGTcaccatcaccatcaccatTGA

Supplementary table1. Primer sequences used for RT-qPCR.

| Gene | Primer | Sequences(5’-3’) |
| --- | --- | --- |
| Mouse gapdh | Forwrd | GGTTGTCTCCTGCGACTTCA |
|  | Reverse | TGGTCCAGGGTTTCTTACTCC |
| Mouse IL-10 | Forwrd | TGCCAAGCCTTATCGGAAATGATCC |
|  | Reverse | AGCCGCATCCTGAGGGTCTTC |
| Mouse IL-6 | Forwrd | CTTCTTGGGACTGATGCTGGTGAC |
|  | Reverse | TCTGTTGGGAGTGGTATCCTCTGTG |
| Mouse IL-1b | Forwrd | CACTACAGGCTCCGAGATGAACAAC |
|  | Reverse | TGTCGTTGCTTGGTTCCTCCTTGTAC |
| Mouse TNF-a | Forwrd | CGCTCTTCTGTCTACTGAACTTCGG |
|  | Reverse | GTGGTTTGTGAGTGTGAGGGTCTG |
| Mouse GLUT1 | Forwrd | GACAAGACACCCGAGGAG |
|  | Reverse | ACTGCTGGCTGAGGAAAG |
| Mouse HIF-1a | Forwrd | TGAACCCATTCCTCATCC |
|  | Reverse | CGGCCCAAAAGTTCTTC |
| Mouse PFKFB3 | Forwrd | GCACTAAAGCCGAGATGTG |
|  | Reverse | CCCGAGTCCAGAAGTCC |
